# TEMPO‐Mediated Paired Electrosynthesis of Ethylene Glycol from Formaldehyde and Methanol at High Current Densities

**DOI:** 10.1002/cssc.202500123

**Published:** 2025-04-11

**Authors:** Valtteri Oksanen, Kiia Malinen, Tao Hu, Alexander Reznichenko, Tom Wirtanen

**Affiliations:** ^1^ Chemical and Polymer Synthesis VTT Technical Research Centre of Finland Ltd., Box 1000 FI‐02044 Espoo Finland; ^2^ School of Chemical Engineering Aalto University Box 11000 FI‐00076 Aalto Finland; ^3^ Research Unit of Sustainable Chemistry University of Oulu Box 4300 FI‐90014 Oulu Finland

**Keywords:** alcohols, aldehydes, c1 building blocks, dimerization, electrochemistry

## Abstract

Herein, a paired electrosynthesis of ethylene glycol from formaldehyde and methanol facilitated by TEMPO is reported. The use of TEMPO accentuates formaldehyde production at the anode, providing additional formaldehyde into the cathodic coupling process. The reaction is performed in water/methanol solution in a simple undivided cell using sulfuric acid‐treated graphite electrodes with industrially feasible current densities between 300 and 350 mA cm^−2^. Other components of the reaction are sodium chloride which is used as a supporting electrolyte and tributylmethylammonium chloride which raises the current efficiency. With a slight modification in the reaction temperature and current density, the outcome can be tuned from high current efficiency toward higher chemical yields. The conditions of the batch reaction are successfully transferred to a continuous flow‐cell arrangement. Mechanistic studies indicate the involvement of hydroxymethyl radicals in the electrolysis, and deuterium‐labeling experiments show the partial conversion of methanol into formaldehyde and ethylene glycol.

## Graphical abstract

A high‐yield paired electrosynthesis of ethylene glycol from formaldehyde and methanol at current densities up to 350 mA cm^−2^ is demonstrated. The presented method utilizes inexpensive graphite electrodes in either batch or flow cell and 2,2,6,6‐tetramethylpiperidin‐1‐yl)oxyl (TEMPO) as a redox mediator. Mechanistic studies indicate toward the formation of hydroxymethyl radicals during the electrolysis.

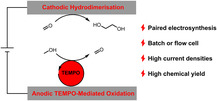

## 1 Introduction

Ethylene glycol (EG) is among the most important commodity chemicals with various uses in different applications either as such as a precursor for other chemicals or constituent in various materials such as polyesters.^[^ ^1^ ^]^ The current industrial synthesis proceeds by epoxidation and subsequent hydration of fossil‐based ethylene in a very carbon‐intensive process, and it has been reported that 4.2 tCO_2_ is produced for every ton of EG manufactured.^[^ ^2^ ^]^ Consequently, this highly detrimental environmental effect is amplified by the high annual ethylene glycol production volume of 42 Mt.^[^ ^2^ ^]^ Therefore, there is an immense potential in the development of sustainable EG synthesis.

**Scheme 1:**
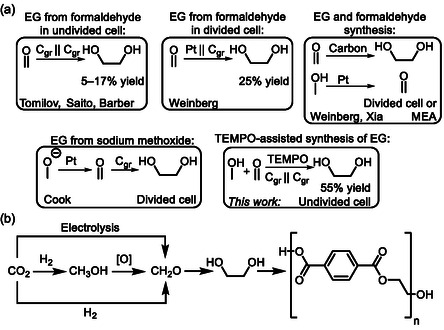
a) Different electrolysis strategies toward ethylene glycol and b) possible value‐chain to fully CO_2_‐based ethylene glycol.

Electrosynthesis has been hallmarked as the 21st‐century technique for organic synthesis due to several aspects that leverage its economic and ecologic benefits in comparison to other synthetic methods.^[^ ^3^, ^4^, ^5^, ^6^, ^7^, ^8^, ^9^, ^10^ ^]^ In this respect, electrosynthesis of EG from formaldehyde via pinacol‐coupling has been also investigated (**Scheme** **1**, top).^[^ ^2^, ^11^, ^12^, ^13^, ^14^, ^15^, ^16^, ^17^, ^18^, ^19^, ^20^ ^]^ Tomilov et al. described EG synthesis from formaldehyde in 1973 using graphite electrodes and a Hg(II) catalyst at 25% current efficiency (c.e.).^[^ ^11^ ^]^ Thereafter, Saito produced EG from alkaline formaldehyde solutions with graphite electrodes in an undivided cell. The highest c.e. of 81% and 5% EG chemical yield was achieved at 50 °C with a current density of 111 mA cm^−2^.^[^ ^12^ ^]^ Barber electrosynthesized EG in neutral or acidic media with c.e. and yield of 83% and 17%, respectively, at 70 °C, using NMe_4_Cl as a supporting electrolyte in an undivided cell with graphite electrodes.^[^ ^13^ ^]^ Higher current efficiencies were achieved in the presence of organic cosolvents such as THF, MeCN, or NMP.^[^ ^13^ ^]^ Weinberg and Mazur reported a detailed study in 1991 on formaldehyde hydrodimerization in which they employed platinum or platinized titanium anodes and graphite cathodes.^[^ ^14^ ^]^ Their best result was obtained in a divided cell at 80 °C using 52% formaldehyde solution which resulted in 25% yield and 83% c.e. of EG, however, when a more dilute solution of 37% formaldehyde was used, the best EG yield obtained was 18% with 80% c.e. Leitmotiv in these earlier studies has been the particular suitability of graphite cathodes in the transformation, whereas other carbon materials, mercury, and lead cathodes have performed inferior.

Paired electrolysis, where both electrode processes contribute toward synthesis meaningfully, can help to maximize the energy, cost, and atom efficiency of electrosynthesis.^[^ ^21^, ^22^, ^23^, ^24^, ^25^, ^26^ ^]^ Consequently, several paired EG electrolysis have been described in the literature (Scheme 1, top). In these reports, the cathodic pinacol‐coupling is complemented with an anodic process where the oxidation of either methanol or sodium methoxide to formaldehyde takes place.^[^ ^2^, ^18^, ^19^ ^]^ Weinberg et al. reported that when a methanol vapor is fed to a high‐surface area carbon gas diffusion anode, that contains a platinum catalyst, methanol is oxidized to formaldehyde and EG is formed simultaneously from formaldehyde at the cathode in a divided cell configuration.^[^ ^18^ ^]^ However, the drawback of the divided cell setup is, among other factors, that the produced formaldehyde is not directly consumed in the cathodic process. More recently, Xia, Jiao et al. described a process for ethylene glycol electrosynthesis and methanol oxidation into formaldehyde using membrane electrode assembly with titanium fiber felt anode deposited with platinum particles and porous carbon paper cathode deposited with carbon black under potentiostatic conditions.^[^ ^2^ ^]^ Another approach has been to oxidize two sodium methoxides at platinum anode to form methoxide radicals which then subsequently form formaldehyde and methanol by radical disproportionation. This step is followed by a cathodic coupling at graphite cathode to EG to form a paired domino process.^[^ ^19^ ^]^ Noteworthy, in both cases, there is an inherent electron mismatch. The anodic oxidation that forms two formaldehydes requires four electrons, whereas the cathodic pinacol‐coupling of the two formaldehydes is a two‐electron process, which leads to a suboptimal use of electricity in the direct domino electrolysis of methanol to EG.

We became interested in EG electrosynthesis from formaldehyde as the reaction can potentially decarbonize high‐volume polyesters such as PET, or its more contemporary alternative PEF, as formaldehyde can be (in)directly produced from carbon dioxide using either catalytic or electroorganic methods (Scheme 1, bottom).^[^ ^27^, ^28^ ^]^ Particularly, we were interested in developing a simple methodology that can be performed in undivided cells in high chemical yields and conversions, which could potentially simplify the downstream separation and recycling, ultimately leading to reduced economic costs of the overall process. Moreover, we wanted to avoid both highly basic reagents such as sodium methoxide and precious metal cathodes or electrocatalysts. Most of the previous reports have focused on achieving high current efficiencies, but in these cases, conversions have been rather low viz. we are only aware of one example where a yield higher than 20% has been reported (see SI for more detailed comparison). To achieve this goal, we hypothesized that an organic redox mediator such as TEMPO,^[^ ^29^, ^30^ ^]^ could augment the methanol electro‐oxidation to formaldehyde and keep its concentration high enough for the EG synthesis to proceed at high conversions.^[^ ^14^ ^]^

## 2 Results and Discussion

We commenced experiments in an undivided batch‐type of cell using isostatic graphite (Sigrafine V2100, SGL Carbon) electrodes. Our objectives for this study were to optimize the reaction conditions for c.e. at lower conversion and then proceed to optimize the yield at high conversion. The electrodes were pretreated with 0.5 m sulfuric acid which was beneficial for c.e. and yield (SI). Pretreatment of graphite electrodes, such as soaking in 5% HCl,^[^ ^14^ ^]^ electro‐oxidation in 10% H_2_SO_4_^[^ ^14^ ^]^ or oxidation in 30% H_2_O_2_^[^ ^19^ ^]^ has been demonstrated to increase current efficiencies in EG synthesis. The effect of the reaction temperature (60–80 °C), current density (250–350 mA cm^−2^), amount of NaCl supporting electrolyte (0.6–1.0 m), NBu_3_MeCl additive (0.08–0.13 m), TEMPO redox mediator (0.02–0.03 m), and the amount of charge (0.6–1 F) were then explored using a two‐level Plackett–Burman experimental design (SI). After this continuous parameter screening, we were able to obtain EG in 28% yield and 94% c.e. (average of three experiments) at 80 °C with 350 mA cm^−2^ current density in the presence of 0.03 m TEMPO, 0.6 m NaCl, and 0.13 m of NBu_3_MeCl. The concentration of the formaldehyde in the electrolysis was 22 wt% which was obtained by diluting a commercial 37 wt% solution with 7:3 MeOH:H_2_O.

**Scheme 2:**
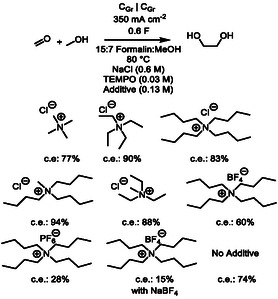
Screening of different quaternary ammonium salts in the presence of TEMPO additive.

Quaternary ammonium cations (QACs) can decorate cathode's surface which can lead to, for example, attenuating of the parasitic hydrogen formation.^[^ ^31^, ^32^ ^]^ Screening of different QACs (NMe_4_Cl, NEt_4_Cl, NBu_4_Cl, and NEt_3_MeCl) showed that NBu_3_MeCl (94% c.e.) was the best performing (**Scheme** **2**). Almost similar c.e. of 90% was obtained with NEt_4_Cl which indicates that there are viable alternatives if the lipophilicity of QAC is a critical parameter in a technical process. Other tested tetra‐alkyl ammonium cations (NEt_3_MeCl, NBu_4_Cl, and NMe_4_Cl) gave lower, but still satisfactory c.e. ranging from 88 to 77%. Conversely, the effect of counter‐anion seems somewhat critical, as NBu_4_BF_4_ (c.e. 60%) and NBu_4_PF_6_ (c.e. 28%) gave 23% and 55% lower c.e. than NBu_4_Cl. Surprisingly, replacing NaCl and NBu_4_Cl with NaBF_4_ and NBu_4_BF_4_ led to a further dramatic decrease of c.e. to 15%. Interestingly, Tomilov et al. have reported that among several tested inorganic and organic potassium‐supporting electrolytes, KCl performed best in their EG synthesis.^[^ ^17^ ^]^ Furthermore, in the absence of QAC, the c.e. was 74%, which clearly demonstrates the positive effect of QAC as an additive in the EG electrosynthesis. Noteworthy, in the presence of sole NBu_3_MeCl, the terminal voltage was too high and an excessive gas formation was observed.

**Figure 1:**
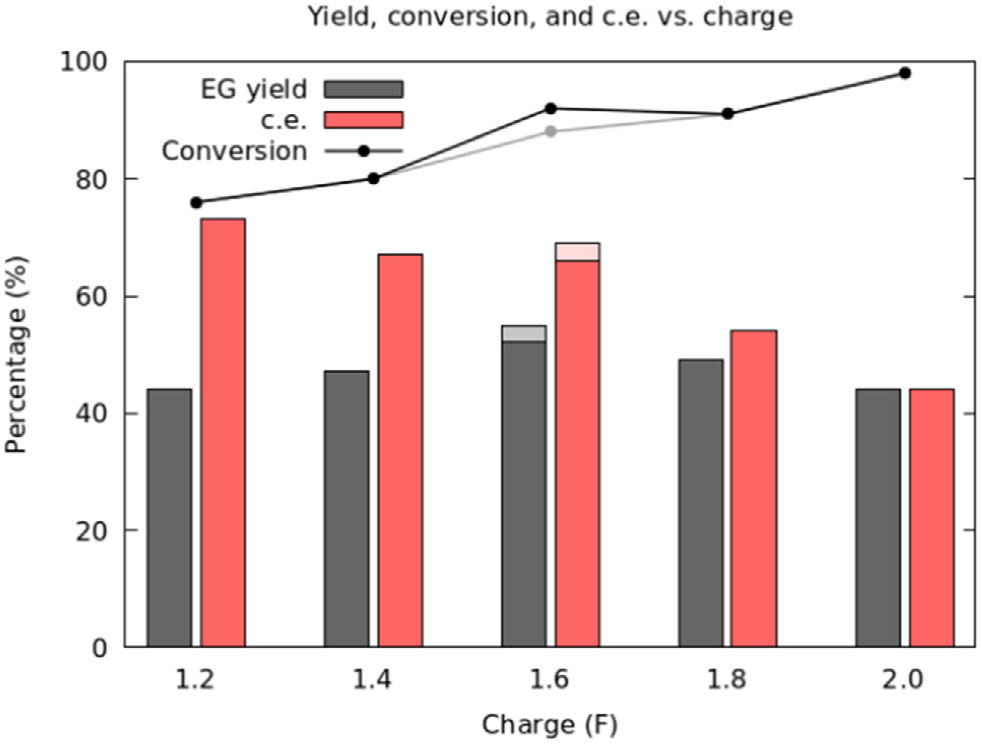
Screening of charge. Optimized conditions at 1.6 F are displayed with lighter colors.

We then proceeded to optimize the yield of EG at high conversions. Screening of the applied charge at 80 °C with 350 mA cm^−2^ between 1.2 and 2 F indicated that the yield is maximized at 1.6 F (**Figure** **1**). With this amount of charge, EG yields were then further optimized with a two‐level full factorial design for three factors using temperature (70–80 °C), the amount of TEMPO (0.03–0.04 m), and current density (250–350 mA cm^−2^) as continuous variables (SI). Gratifyingly, we obtained 55% yield with 69% c.e. at 88% conversion at a thrice replicated center point (75 °C, 0.03 m of TEMPO, 300 mA cm^−2^) which indicates that the reaction conditions, with the constrained variables, are close to an optimum. The reaction could also be scaled‐up from 200 to 320 and 640 mmol by recirculating the electrolyte solution in a flow‐cell. In these cases, yields between 46 and 51% and c.e. between 58 and 63% were obtained depending on the scale and flow‐rate in the flow electrolysis (for further details, see Supporting Information).

Control studies were then performed for uncovering additional insights for the overall process (**Table** **1**). The addition of TEMPO is beneficial with the both reaction conditions (Entries 1–4). Moreover, the reaction does not proceed in the absence of current, yet some formaldehyde is lost either due to evaporation or chemical reactions (Entry 5). Omitting formaldehyde is highly detrimental for the production of EG, and only 1% c.e. is obtained in this case (Entry 6). This is in line with the previous report that discusses the importance of formaldehyde concentration toward high current efficiencies of EG.^[^ ^14^ ^]^ Furthermore, in the absence of added formaldehyde, c.e. for electrolytically produced formaldehyde is 28% and 10% in the presence and absence of TEMPO (Entries 6–7), respectively, which indicates that TEMPO facilitates the formaldehyde production. Replacing 7:3 MeOH:H_2_O with 7:3 MeOH:phosphate‐buffer at pH of 5.8, 6.0, and 6.4 did not improve yields further (Entries 8–10). The discrepancy between the conversions and EG yields (e.g., Entry 3) is mainly explained by the conversion in the absence of current (Entry 5), possibly due to evaporation or thermal formaldehyde side‐reactions. Possible electrolytic side‐reactions affecting c.e. include cathodic reduction of formaldehyde into methanol,^[^ ^2^ ^]^ and formation of propylene glycol.^[^ ^7^ ^]^ The latter was also detected with ^1^H nuclear magnetic resonance (NMR) from Entries 1–4 in trace amounts. The mass of the gasses produced during electrolysis (≈0.4 g) was small compared to the overall mass of the reaction. Furthermore, alternative formaldehyde sources 1,3,5‐trioxane and paraformaldehyde failed to yield EG.

**Table 1:** Control studies in the EG electrosynthesis.

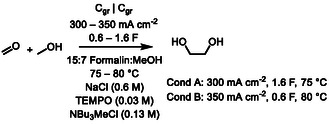				
Entry	Modifications to standard conditions (A/B)	EG yield (%)	Conversion (%)	c.e. (%)c)
1	(A)a)	55	88	69
2	w/o TEMPO (A)b)	41	96	52
3	(B)a)	28	44	94
4	w/o TEMPO (B)a)	23	61	76
5	No electricity (B)	0	11	–
6	No formaldehyde with TEMPO (B)	<1	–	1 (28)
7	No formaldehyde w/o TEMPO (B)	0	–	0 (11)
8	Phosphate‐buffer (pH 5.8) instead of H_2_O (B)	25	47	84
9	Phosphate‐buffer (pH 6.0) instead of H_2_O (B)	24	46	78
10	Phosphate‐buffer (pH 6.4) instead of H_2_O (B)	22	48	74

a) Average of three replicates.

b) Average of two replicates.

c) Current efficiency for formaldehyde in parenthesis.

**Scheme 3:**
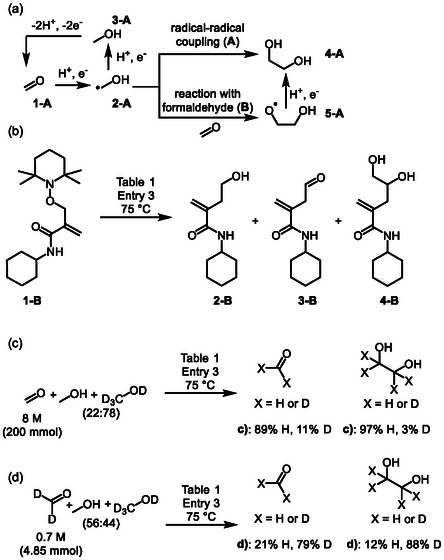
a) Tentative reaction pathways. b) Mechanistic studies with **1**‐**B** radical trap. Masses matching the structures displayed were detected with HPLC/HR‐ESI‐MS. c,d) Key results from deuteration experiments.

The reaction mixture was also analyzed with high‐performance liquid chromatography (HPLC) using high‐resolution electrospray ionization mass spectrometry (HR‐ESI‐MS) as detector for identifying possible radical intermediates that are formed during the electrolysis. For this purpose, we employed a recently described radical trap (**Scheme** **3**, middle, **1‐B**), which has been shown to be resistant toward false positives formed via nonradical pathways.^[^ ^33^ ^]^

HR‐ESI‐MS measurements from this mixture displayed a mass matching for the hydroxymethyl radical addition compound (Scheme 3, middle, **2‐B**). Interestingly, hydroxymethyl radical has been proposed as a probable intermediate in EG synthesis from formaldehyde based on isotopic‐labeling studies and periodic density functional theory calculations.^[^ ^2^ ^]^ Moreover, the desorption energies of the hydroxymethyl radical from carbon, copper, and palladium surfaces rationalized the particular suitability of the carbon‐based cathodes in the EG synthesis.^[^ ^2^ ^]^ The detected intermediate supports the literature hypothesis, however, we cannot currently rule out that the detected species is not formed from an anodic process instead of cathodic one. In addition to **2‐B**, traces of **3‐B** and **4‐B** were also detected. We speculate that **2‐B** is first formed, which then subsequently could be oxidized to **3‐B**, possibly indicating that the detected m/z is derived from hydroxymethyl radical **2‐A** (i.e., not from methoxy radical). Furthermore, **4‐B** could be an intermediate toward propylene glycol (vide supra), that is known to form from glycerol.^[^ ^34^ ^]^ Based on the literature and HR‐ESI‐MS measurements, a tentative reaction pathway for EG formation is depicted in Scheme 3. Formaldehyde (**1‐A**) is first reduced to a hydroxymethyl radical (**2‐A**), that then either can be reduced to a methanol (**3‐A**),^[^ ^2^ ^]^ react with an another hydroxymethyl radical,^[^ ^2^ ^]^ or with a free formaldehyde.^[^ ^35^ ^]^ The former pathway directly furnishes ethylene glycol (**4‐A**), whereas the latter leads to an O‐centered radical intermediate (**5‐A**), which is then further reduced to EG (**4‐A**). Intermediate **5‐A** could also be a part in a radical chain,^[^ ^35^ ^]^ but mechanistic studies by Xia, Jiao et al. with ^13^C‐labeled methanol have shown that the methanol is not incorporated in the EG in divided cell.^[^ ^2^ ^]^

Further mechanistic experiments were then conducted for inspecting the hypothesized paired process and the role of TEMPO in it (Scheme 3c–d). The control experiments (Table 1) clearly show more formaldehyde in the presence of TEMPO after the reaction than without it (Entry 1 vs 2; Entry 3 vs 4, Table S11, Supporting Information). Furthermore, formaldehyde current efficiency is higher with TEMPO than in its absence (Table 1, Entry 5 vs 6) when no formaldehyde is added into the reaction mixture. As the former is an indirect indication of a paired process, and the latter result is obtained in modified reaction conditions, we wanted to scrutinize this hypothesis further. The synthesis was then conducted with deuterated methanol and formaldehyde (Scheme 3c–d). These results show that when mixtures of formaldehyde, CD_3_OD and CH_3_OH, or deuterated formaldehyde, CH_3_OH and CD_3_OD are electrolyzed, a part of the CH_2_ or CD_2_ groups in EG and formaldehyde originate from (deuterated) methanol, demonstrating that anodically generated formaldehyde is incorporated partially into ethylene glycol. Based on these combined results, TEMPO seems to augment the anodic oxidation of methanol to formaldehyde, but it is difficult to assess the magnitude of this effect in the EG synthesis due to several complicating factors (please see further discussion in the supporting information).

The effect of the sulfuric acid treatment on the graphite cathode was then investigated with X‐ray photoelectron microscopy (XPS), inductively‐coupled mass spectrometry (ICP‐MS), and inductively‐coupled optical emission spectroscopy (ICP‐OES). Based on the literature, we reasoned that the beneficial effect of the sulfuric acid treatment of the graphite electrodes could originate from at least two scenarios. First, Weinberg et al. have postulated that the involvement of surface oxygen groups can explain the high performance of the graphite cathode,^[^ ^9^ ^]^ while on the contrary, Xia, Jiao et al. showed that with carbon black catalyst supported on a graphite paper cathode, the current efficiencies for ethylene glycol decreased when the amount of different surface oxygen groups increased.^[^ ^2^ ^]^ Second, several metal impurities such as Ca, Fe, and Cu are known to inhibit the electrolysis at low concentrations.^[^ ^14^, ^17^ ^]^ Our XPS measurements from the untreated and sulfuric acid‐treated graphite electrodes reveal only a minor increase in the total surface oxygen content from 1.8% to 2.0%, while the relative distributions of the oxygen species are very similar (SI). Comparable behavior in respect to the surface oxygen content has been reported for another type of graphite, which was treated with 20% H_2_SO_4_ solution at 70 °C.^[^ ^36^ ^]^ In contrast, ICP‐MS and ICP‐OES analysis of the sulfuric acid used in the pretreatment of the electrodes display the removal of known inhibitors such as Fe and Ca (SI). Currently, the removal of minute amounts of impurities from the cathode seems a more sound explanation, however, there might be also other factors that contribute to the effect.

## 3 Conclusions

In this communication, we demonstrated that the addition of TEMPO redox mediator elevates the current efficiency and yields of formaldehyde electrolysis to ethylene glycol at high industrially relevant current densities. Based on the experimental evidence, we propose that the addition of TEMPO enables methanol oxidation to formaldehyde contributing toward a more successful pairing of the anodic and cathodic half‐reactions. We also demonstrated that the optimized batch reaction conditions can be transferred successfully into a flow‐cell, which paves the way for the future development of a technical process toward fully CO_2_‐based ethylene glycol in undivided cells.

## Supporting Information

The authors have cited additional references within the Supporting Information.^[37–48]^

## Conflict of Interest

The authors declare no conflict of interest.

## Author Contributions

**Tom Wirtanen**: conceptualization (equal); investigation (supporting); methodology (equal); Supervision (lead); writing—original draft (equal); writing—review & editing (equal). **Valtteri Oksanen** conceptualization (equal); investigation (lead); methodology (equal); writing—original draft (equal); writing—review & editing (equal). **Kiia Malinen**: investigation (supporting). **Tao Hu**: investigation (supporting). **Alexander Reznichenko**: supervision (supporting); writing—review & editing (supporting).

## Supporting information

Supplementary Material

## Data Availability

The data that support the findings of this study are available from the corresponding author upon reasonable request.
