# Supplementary material for: TEMPO‐Mediated Paired Electrosynthesis of Ethylene Glycol from Formaldehyde and Methanol at High Current Densities
Source: ChemSusChem. 2025 Apr 11;18(12):e202500123. doi: 10.1002/cssc.202500123 (PMC12175047; doi:10.1002/cssc.202500123)
Supplement: Supplementary file 1 — Supplementary Material [file CSSC-18-e202500123-s001.pdf]

Contents

EXPERIMENTAL 2

1. General Information **2**

2. Quantification of Formaldehyde and Ethylene Glycol from Electrolysis Solutions **3**

3. Standard Operation Conditions in Electrolysis Experiments **4**

4. Screening of Reaction Conditions **5**
4.1. Preliminary Screening 5
4.2. The Effect of Sulfuric Acid Pretreatment of Graphite Electrodes 6
4.3. The Optimization of the Current Efficiency at Lower Conversion 6
4.4. Screening of Different Electrodes 8
4.5. Screening of Redox Mediators 9
4.6. The Optimization of EG Yield in Higher Conversion 10
4.7. Flow-Electrolysis 11

5. Comparison of the Literature Reported Values with the Current Protocol **12**

6. Inductively-Coupled Mass Spectrometry (ICP-MS) and Inductively-Coupled Optical Emission Spectroscopy (ICP-OES) Measurements **13**

7. X-ray Photoelectron Spectroscopy (XPS) **14**

8. Trapping Experiments **17**

9. Mechanistic Experiments with Methanol-d_4 and Formaldehyde-d_2 **18**
9.1 Electrosynthesis of Formaldehyde-d_2. 21
9.2 Characterization of ^{2}H-Labelled Compounds 22

10. Synthesis of CHANT **22**

11. NMR-traces **24**

12. HPLC / ESI-HR-MS traces **29**

13. References **32**

Experimental

1. General Information

Chemicals: All chemicals and solvents were purchased and used without any purifications unless otherwise stated. Analytical grade formaldehyde was obtained from Sigma-Aldrich as a 37 wt-% water solution, stabilized with 10% MeOH. Water was purified with Milli-Q water purification system (Milli-Q IQ 7000, Merck)

Electrodes: Graphite electrodes (C_{gr}, Sigrafine V2100) were purchased from SGL Carbon. Prior to use, the electrodes were placed in a 0.5 M H_2SO_4 solution for ten minutes and subsequently rinsed with water and acetone. Graphite surfaces were cleaned after reactions with a synthetic polyamide fiber polishing pad, after which they were sonicated for around ten minutes in a water bath, further washed with water and acetone, and finally polished with a paper towel and acetone. The glassy carbon (GC, Sigradur G, HTW Hochtemperatur-Werkstoffe GmbH) surface was washed with acetone and water, and wiped with a paper towel prior to use. Boron-doped diamond electrodes (BDD, Merck) were conditioned by electrolyzing in 20% H_2SO_4 with BDD as an anode and C_{Gr}-plate as a cathode (10 C/cm², 10 mA/cm²) with subsequent rinsing with water and acetone.[1] Electrode dimensions were 2 x 6 cm and the interelectrode gap was 0.5 cm in batch reactions. In flow electrolysis, the electrode area was 10 cm^2 and the interelectrode gap 0.4 cm.

Electrolysis: All the batch electrolysis experiments were carried out in custom-manufactured 25 mL glass cells with the diameter and height of the reactor part being 3 and 7 cm, respectively (Figure S1, left). The cell is a smaller version of a commercially available SynLectro (Merck) glass cell developed by Waldvogel research group. Electrode holders and PTFE stoppers were obtained from Merck. Flow-electrolysis was performed in ElectroCell Micro Flow Cell. The electrodes in the flow-cell were heated from the backside with a heating thermostat (Lauda CS 6-D) using distilled water as the heat exchanger working fluid (Figure S1, right). The electrolyte solution was pumped with Simdos O2 Dosing Pump (KNF), fitted with a PTFE pump head, into the flow-cell through a coiled PTFE-tube that was heated with a spiral heater (Holroyd). The flow-cell, PTFE-tubing, and spiral heating tape were thermally isolated with a glass fiber fabric. Electrolysis was powered with a four-channel power supply (Rohde & Schwarz HMP4040).

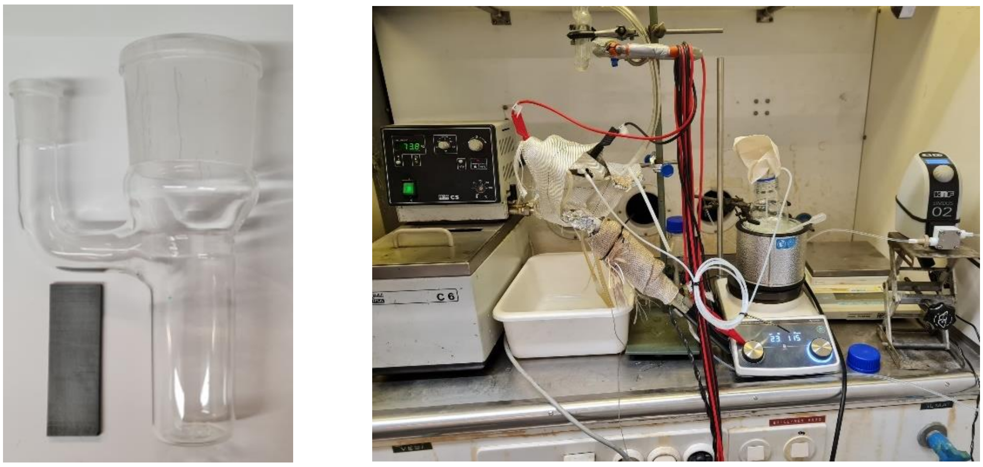

Figure S1. Photographs of the electrochemical batch cell (left), and flow-electrolysis setup (right) used in the study.

2. Quantification of Formaldehyde and Ethylene Glycol from Electrolysis Solutions

Reaction Quantification: NMR spectroscopy was used to monitor the conversion, yield, and selectivity of the reactions. All the spectra were acquired in D_2O with Bruker Avance III 500 MHz Ultrashield spectrometer and referenced to methyl sulfone ($DMSO_2$, 3.06 ppm). Formaldehyde was quantified with ^{1}H NMR according to a modified literature method that utilizes formaldehyde-bisulfite adduct.[2] Ethylene glycol was quantified from the same sample. First, the reaction mixture was diluted to 25.0 mL after electrolysis. Second, 50 µl aliquot was drawn and 250 µl of sodium bisulphite (38-40 % in H_2O) and 250 µl D_2O were added into the aliquot. This mixture was aged between 20 to 30 min, after which 50 µl of methyl sulfone solution ($DMSO_2$, 14.6 mg ml^{-1}) in D_2O was added as an internal standard. Third, 50 µl aliquot was further diluted with 550 µl of D_2O, and the mixture was aged further for two hours. ^{1}H NMR spectra (Figure S2) were then recorded by accumulating 12 transients with a relaxation delay of 25 s with a water-suppressed pulse sequence (noesygppr1d). The spectra were processed using MestReNova program (version 14.3.3). The standard processing of the spectra included phase correction, baseline correction (Whittaker Smoother) and referencing to the $DMSO_2$ singlet at 3.06 ppm. $DMSO_2$ resonance was then integrated from 3.08 to 3.04 ppm, ethylene glycol (3.58 ppm, s, 4H) from 3.61 to 3.55 ppm and formaldehyde-bisulfite adduct (4.31 ppm, s, 2H) from 4.34 to 4.29 ppm. Molar amounts of formaldehyde and ethylene glycol were then calculated and converted into conversion and yield.

The current efficiency was calculated by dividing the amount of formed ethylene glycol by the theoretical maximum amount of ethylene glycol that could be generated during electrolysis with constant current (Formula S1). The number of electrons required to generate one molecule of ethylene glycol is 2. Thus, the theoretical maximum amount of ethylene glycol is $\frac{1}{2} \times n(e^-)$.

Formula S1. Calculation of the current efficiency.

$$c.e. = \frac{n(EG)}{\frac{1}{2} \times n(e^-)} \times 100\% = \frac{n(EG)}{\frac{1q}{2q(e^-) \times N_A}} \times 100\% = \frac{2q(e^-) \times N_A \times n(EG)}{I \times t} \times 100\%$$

where $q(e^-)$ is charge of an electron, q electric charge passed during electrolysis, N_A is Avogardo's constant, $n(EG)$ is the molar amount of produced ethylene glycol, I is electrolysis current in amperes, and t is electrolysis time in seconds.

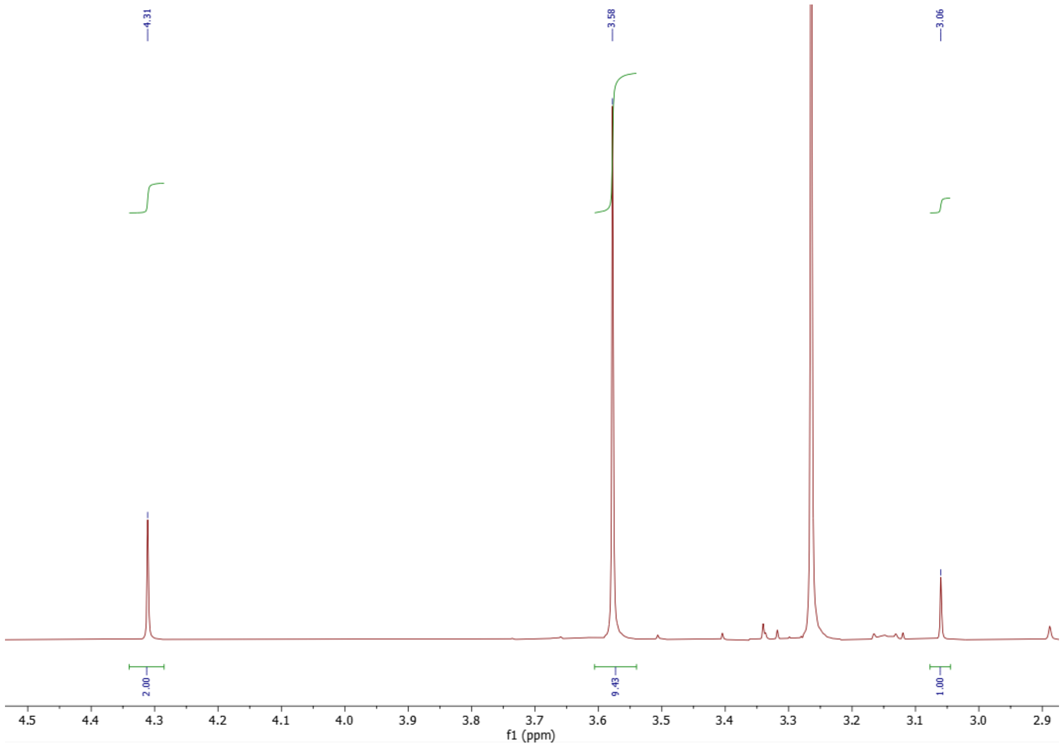

Figure S2. A representative example of NMR spectrum used to determine ethylene glycol (3.58 ppm, s, 4H) and formaldehyde bisulfite-adduct (4.31 ppm, s, 2H) concentrations from reaction mixtures using $DMSO_2$ (3.06 ppm, s, 6H) as an internal standard.

3. Standard Operation Conditions in Electrolysis Experiments

Sodium chloride, a redox mediator and a quaternary ammonium salt were weighted into a glass cell (diameter 3 cm, height 7 cm) equipped with a magnetic stirring bar (diameter 3 mm, length 10 mm). 15 mL 37 wt-% formaldehyde solution was then measured into the cell using a measuring burette, followed by 7 mL MeOH and optionally 3 mL H_2O. A condenser with a water circulation and electrodes in a PTFE holder were attached to the cell. The depth and area of electrodes immersed in the electrolyte were 3.4 cm and 6.8 cm^2 respectively. The cell was immersed in a silicone oil bath, magnetically stirred at 300 rpm, and preheated for 15 minutes at the desired temperature. The reaction temperature refers to the temperature of the silicone oil bath. After the preheating, a constant current was passed through the electrodes for a predetermined

amount of charge. After the completion of the electrolysis, the mixture was removed from the oil bath and analyzed with the standard quantification method (Section 2).

Flow electrolysis was performed in an ElectroCell Micro Flow Cell (10 cm^2 electrode area, 4 mm electrode gap). In these experiments, a pre-heated electrolyte solution was pumped at a rate of 10 or 20 mL/min into a heated flow cell which was operated at 75 °C and 300 mA cm^{-2}. The reaction temperature refers to the temperature of the heating thermostat. The solution was recirculated in the electrolyser until the desired charge of 1.6 F was passed. After the completion of the electrolysis, the mixture was analyzed with the standard quantification method (Section 2).

4. Screening of Reaction Conditions

4.1. Preliminary Screening

Preliminary screenings (Table S1) were conducted with graphite electrodes in solutions consisting of 15 mL 37 wt-% formaldehyde solution and 7 mL MeOH with NaCl (1.2 g, 20.5 mmol), TEMPO (0, 0.1 or 0.2 g, 0.64 or 1.3 mmol) and tributylmethylammonium chloride (0.6 g, 2.5 mmol). In Entry 6, an additional 3 mL water was added to the solution. Selected examples of these studies are presented below in Table S1. Entry 6 is reported as an average of three replicates. Shortly, in the preliminary screenings, we found that TEMPO increases both ethylene glycol yields, and current efficiencies, and we therefore decided to include TEMPO in further optimizations. A high current density (300 mA cm^{-2}) and temperature (70 °C) were also favorable and chosen as the starting values for further optimizations. The electrodes used in the preliminary screening were not treated with sulfuric acid.

Table S1. Examples from preliminary screening of ethylene glycol electrosynthesis.

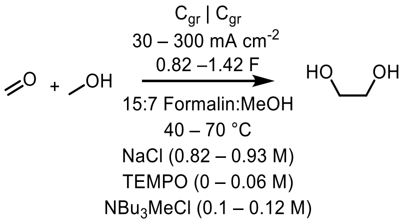

Entry	Charge (F)	j (mA cm^{-2})	T (°C)	TEMPO (mg)	yield (%)[a]	c.e. (%)
1	1.38	300	70	0	33	49
2	1.41	300	70	100	41	58
3	1.32	300	70	200	43	66
4	1.28	30	70	100	15	22
5	1.32	300	40	100	10	15
6	0.82	300	70	100	25	60

[a] Yields determined by ^{1}H NMR using dimethyl sulfone ($DMSO_2$) as an internal standard.

4.2. The Effect of Sulfuric Acid Pretreatment of Graphite Electrodes

To study the effect of sulfuric acid pretreatment of the graphite electrodes, three replicate electrolysis with untreated graphite electrodes were compared to three replicates with sulfuric acid treated electrodes (Table S2). The sulfuric acid treatment was performed by placing the electrodes in a 0.5 M H_2SO_4 solution for ten minutes after which they were rinsed with water and acetone. All the electrolysis were conducted with reaction parameters obtained from the preliminary screening (Table S1, Entry 6). We observed that both the yield and current efficiencies are improved when sulfuric acid treated electrodes are used in the electrolysis.

Table S2. The effect of sulfuric acid treatment on the yield and current efficiency across three replicates.

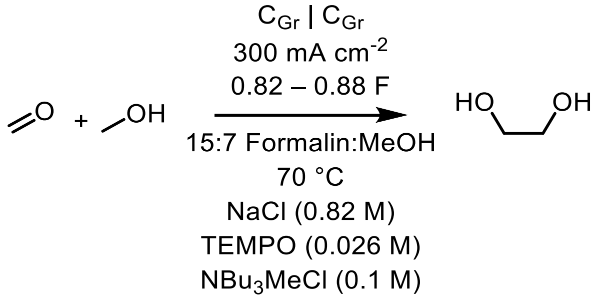

**untreated graphite**		**sulfuric acid treated graphite**	
yield (%)[a]	*c.e. (%)*	*yield (%)*[a]	*c.e. (%)*
25	62	38	86
24	60	38	86
23	58	35	85

[a] Yields determined by ^{1}H NMR using dimethyl sulfone ($DMSO_2$) as an internal standard.

4.3. The Optimization of the Current Efficiency at Lower Conversion

The most important parameters for the current efficiency and yield in the electrosynthesis of ethylene glycol were determined with a two-level Plackett-Burman Design of Experiment (DoE) with charge, current density, temperature and amounts of TEMPO, sodium chloride and tributylmethylammonium chloride chosen as continuous parameters (Table S3). The electrolysis was conducted using sulfuric acid pretreated graphite electrodes in a solution that contained 15 mL 37 wt-% formaldehyde, 7 mL MeOH, and 3 mL H_2O with the rest of the molar amounts of TEMPO, NaCl, and tributylmethylammonium chloride determined by the experimental design. The applied charge corresponds to either 30%, 40% or 50% completion of the electrolysis.

The following observations were made on the effects of the different screened parameters on the yield. The applied charge, which directly correlates with the completion degree of the electrolysis, was the most important parameter for increasing the yield of EG. Additionally, increasing the amount of TEMPO improved EG yield. On the contrary, the applied charge had the opposite effect on the current efficiency, and the highest current efficiencies were received with a lower applied charge. Furthermore, increasing the amount of TEMPO from 80 to 120 mg increased the current efficiency. An increase in the current efficiency was observed by increasing temperature and current density, although these two parameters were not statistically significant in the obtained DoE model (Figure S3), which was not refined further, as 94% c.e. was obtained using conditions of Table S3, Entry 4.

Table S3. Optimization of continuous parameters *via* Plackett-Burman Design of Experiment (DoE).

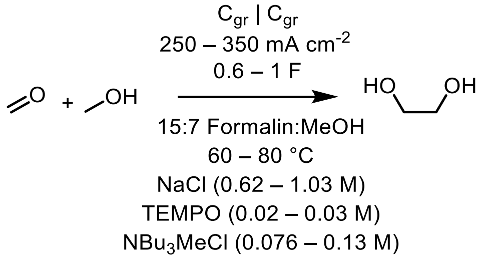

Entry	Charge (C)	j (mA cm^{-2})	T (°C)	NaCl (mg)	TEMPO (mg)	NBu_3MeCl (mg)	Yield (%)[a]	c.e. (%)
1	5789	350	80	900	80	450	25	82
2	9649	350	60	900	120	450	36	71
3	9649	250	80	1500	80	450	32	64
4	5789	350	80	900	120	750	28	94
5	9649	350	60	1500	120	450	39	78
6	9649	350	80	1500	80	750	36	72
7	9649	250	80	900	120	750	40	79
8	5789	250	80	1500	120	450	24	80
9	5789	250	60	1500	120	750	23	75
10	5789	350	60	1500	80	750	22	73
11	9649	250	60	900	80	750	33	65
12	5789	250	60	900	80	450	24	78
13	7718	300	70	1200	100	600	35	85
14	7718	300	70	1200	100	600	32	81
15	7718	300	70	1200	100	600	33	84

[a] Yields determined by ^1H NMR using dimethyl sulfone ($DMSO_2$) as an internal standard.

Figure S3. Results of two-level Plackett-Burman screening of the important parameters. (J = current density, Q = charge, T = temperature, TEMP = amount of TEMPO, NaC = amount of NaCl, s.e = amount of NBu_3MeCl.).

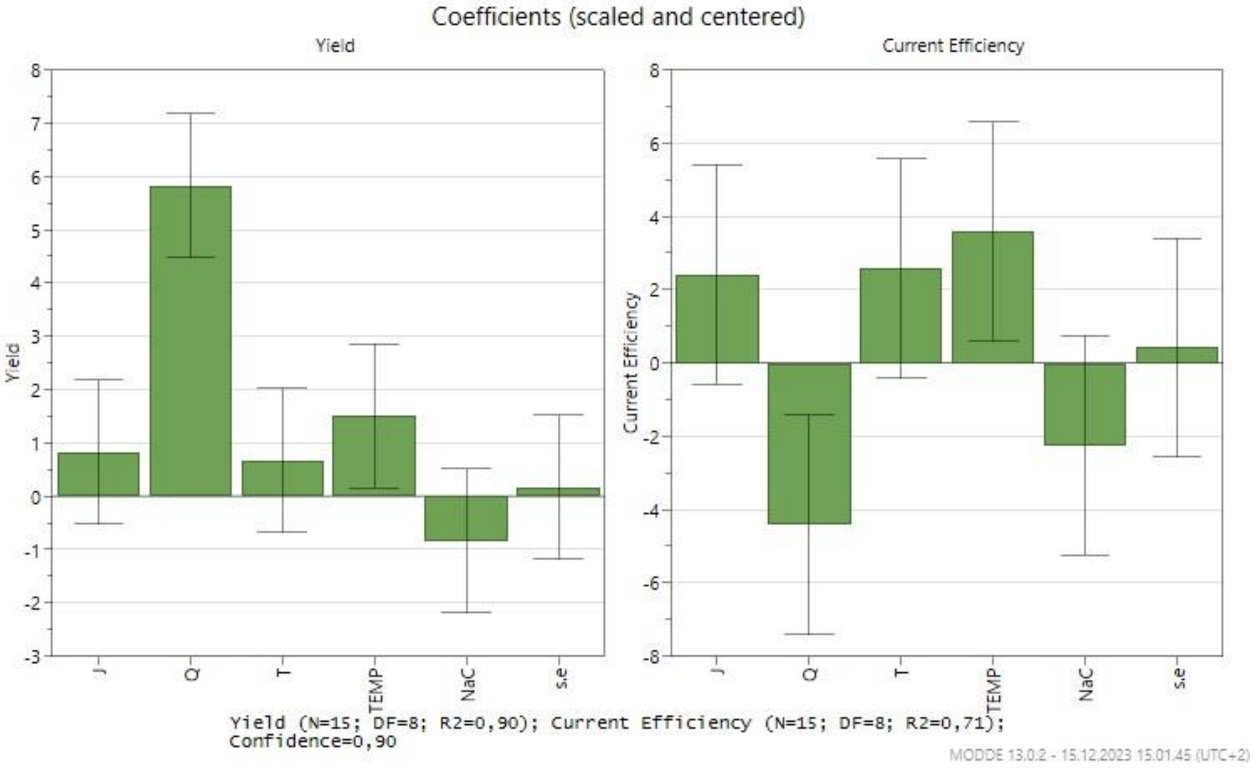

4.4. Screening of Different Electrodes

Previous studies have indicated that carbon-based cathodes are superior to metal-based cathodes in the cathodic coupling of formaldehyde to EG (Table S4). Therefore, we decided to screen different carbon-based cathodes using the optimal conditions established with graphite cathode (Table S3, Entry 4 and Table S4, Entry 1). The alternative cathodes gave only very poor yields and c.e. for ethylene glycol (Entries 2 – 4). In addition, intense gas formation was observed with all alternative cathodes. Interestingly, replacing graphite anode to a glassy carbon or boron-doped diamond (BDD) gave slightly lower current efficiencies of 79 and 71%, respectively.

Table S4. Effect of anode and cathode for paired ethylene glycol formation

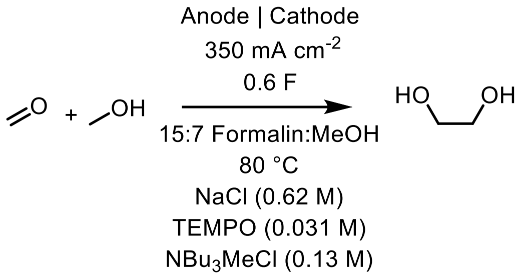

Entry	Anode	Cathode	Conversion (%)[a]	Yield (%)[a]	c.e. (%)
1[c]	Graphite[b]	Graphite[b]	44	28	94
2	Graphite[b]	Graphite felt	44	2	7
3	Graphite[b]	Glassy carbon	39	2	5
4	Graphite[b]	BDD	52	<1	1
5	Glassy carbon	Graphite[b]	49	24	79
6	BDD	Graphite[b]	40	22	71

[a] Yields and conversions determined by ^{1}H NMR using dimethyl sulfone ($DMSO_2$) as an internal standard. [b] Graphite cathodes were treated for ten minutes in 0.5 M H_2SO_4 prior the reaction [c] Average of three experiments

4.5. Screening of Redox Mediators

The effect of different redox mediators was also investigated (Scheme S1), using reaction conditions established during the optimization of current efficiency with TEMPO as the mediator (Table S3, Entry 4). Interestingly, unsubstituted TEMPO provided highest c.e. of 94%, whereas other TEMPO-derivatives such as 4-acetamido-TEMPO, 4-hydroxy-TEMPO, and 4-methoxy-TEMPO gave current efficiencies close to the baseline that was determined without additive to 74% c.e. Alternatively, both cyanuric acid and sodium iodide mediators improved c.e. to 79 and 81%, respectively. Furthermore, the use of sodium bromide instead

of sodium chloride gives slightly improved c.e. of 76%. In contrast to other tested mediators, the use of *N*-hydroxyphthalimide or *N*-hydroxysuccinimide reduced c.e. to 43% and 54% respectively.

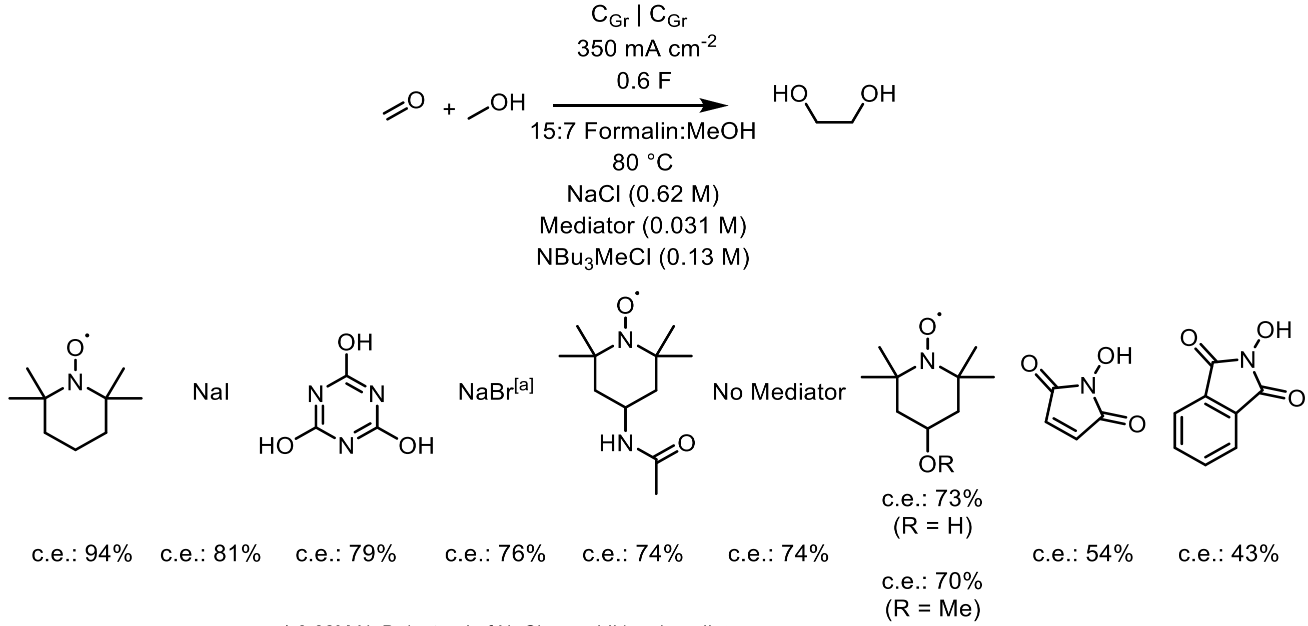

a) 0.62M NaBr instead of NaCl, no additional mediator

Scheme S1. Screening of different redox mediators.

4.6. The Optimization of EG Yield in Higher Conversion

Further optimization of the yield was conducted in higher conversions after having optimized the conditions for current efficiency with 0.6 F (Subsection 4.3) and screening of the applied charge between 1.2 to 2 F (Main Body, Figure 1; Table S5). For this, we conducted electrolysis with 1.6 F using a two-level full factorial design with temperature (70 – 80 °C), the amount of TEMPO (0.03 – 0.04 M), and current density (250 – 350 mA cm^{-2}) as the continuous variables (Table S6). The charge (1.6 F), amount of NaCl (900 mg, 15.4 mmol) and NBu_3MeCl (750 mg, 3.18 mmol), and volumes of formalin (15 mL), MeOH (7 mL) and water (3 mL) were kept constant. The highest yield for EG was obtained at the center point of the full-factorial design (Table S6, Entries 9 – 11).

Table S5. Screening of charge with optimized conditions for current efficiency in the EG electrosynthesis.

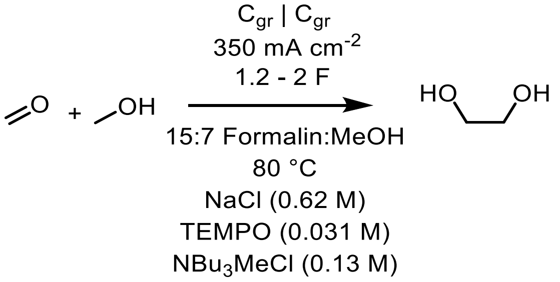

Entry	Charge/Completion	conversion (%)[a]	yield (%)[a]	c.e. (%)
1	1.2 F/ 60%	76	44	73
2	1.4 F/ 70%	80	47	67
3	1.6 F/ 80%	92	52	66
4	1.8 F/ 90%	91	49	54
5	2 F/ 100%	98	44	44

[a] Yields and conversions determined by ^{1}H NMR using dimethyl sulfone ($DMSO_2$) as an internal standard.

Table S6. Optimization of continuous parameters *via* two-level full-factorial Design of Experiment (DoE).

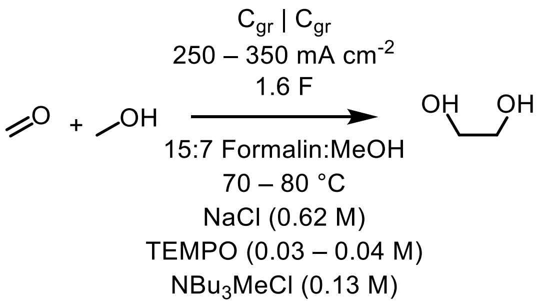

Entry	T (°C)	TEMPO (mg)	j (mA cm^{-2})	yield (%)[a]	c.e. (%)
1	70	100	250	46	58
2	80	100	250	46	58
3	70	140	250	49	61
4	80	140	250	47	58
5	70	100	350	49	61
6	80	100	350	52	64
7	70	140	350	51	64
8	80	140	350	45	57
9	75	120	300	55	68
10	75	120	300	55	69
11	75	120	300	55	69

[a] Yields determined by ^{1}H NMR using dimethyl sulfone ($DMSO_2$) as an internal standard.

4.7. Flow-Electrolysis

The scalability of the method was studied with flow-electrolysis, by taking optimal conditions directly from optimized batch conditions. (Table S6, Entries 9 – 11). Ethylene glycol was obtained in 47 and 46% yields when 320 and 640 mmol formaldehyde were used with 20 ml min^{-1} flow-rate, respectively (Table S7, Entries

1 – 2). A smaller flow of 10 ml min^{-1} resulted in a slightly higher yield of 51%, but the reaction was problematic to conduct due to the gas formation at the end of the reaction (Entry 3).

Table S7. Flow-electrolysis results.

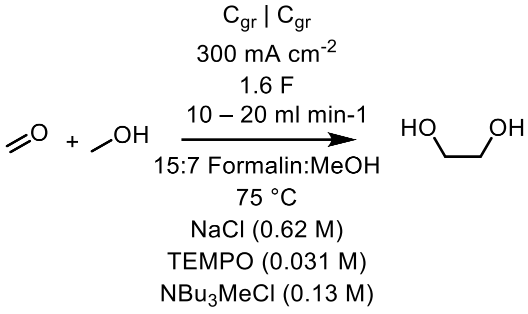

Entry	Formaldehyde (mmol)	Flowrate (ml min^{-1})	conversion (%)[a]	yield (%)[a]	c.e. (%)
1	320	20	81	47	59
2	640	20	85	46	58
3	320	10	90	51	63

[a] Yields and conversions determined by ^{1}H NMR using dimethyl sulfone ($DMSO_2$) as an internal standard.

5. Comparison of the Literature Reported Values with the Current Protocol

Several research groups have studied the dehydrodimerization of formaldehyde into ethylene glycol with graphite cathodes. These studies have mostly focused on maximizing current efficiency, and a chemical yield higher than 20% has been achieved, according to our best knowledge, only once before. In this report, a concentrated formaldehyde solution of 52% was used (Table S8, Weinberg (1), Entry F3 A4). We have compiled a condensed overview of the literature reported data from academic journals and patent literature on the EG electrosynthesis using graphite cathodes as electrodes (Table S8). Noteworthy, several parameters (e.g., cells, possible cell-dividers, anodes, supporting electrolytes, mode of operation) vary between the different entries which makes the direct comparison of the entries unfeasible.

Table S8. Condensed literature reported data from academic journals and patent literature on the EG electrosynthesis with graphite cathodes.

Reference[a]	Entry[b]	FA (mol)	EG (mmol)	Charge (C)	c.d. (mA cm^{-2})	Temp (C)	EG yield (%)	EG c.e. (%)	Completion (%)
Weinberg (1)	T2 E1	1.43	31.0	6050	100	80	4	99	4
"	T2 E2	1.43	31.7	6789	100	90	4	90	5
"	T2 E3	1.43	62.4	12041	200	90	9	100	9
"	T2 E4	1.43	62.3	13350	200	90	9	90	10
"	T2 E5	1.43	77.3	15371	400	80	11	97	11
" (No QAC)	T2 E6	1.43	66.3	15052	400	90	9	85	11

"	T2 E7	1.43	82.7	16625	500	80	12	96	12
"	T2 E8	1.43	73.9	15500	500	90	10	92	11
"	T2 E9	1.43	34.1	7233	1000	90	5	91	5
" (* = 52%)	F3 *A1**	1.89	1.34	259	300	80	0.1	100	0
"	F3 *A2**	1.89	155.9	30129	300	80	17	100	17
"	F3 *A3**	1.89	185.8	49138	300	80	20	73	27
"	F3 *A4**	1.89	232.4	54181	300	80	25	83	30
"	F3 *B1*	1.42	0.67	129	200	80	0.1	100	0
"	F3 *B2*	1.42	58.1	11250	200	80	8	100	8
"	F3 *B3*	1.42	82.1	16293	200	80	12	97	12
"	F3 *B4*	1.42	124.7	30000	200	80	18	80	22
"	F3 *B5*	1.42	128.4	45000	200	80	18	55	33
"	F3 *C1*	1.42	0.7	129	750	80	0.1	100	0
"	F3 *C2*	1.42	72.2	16164	750	80	10	86	12
"	F3 *C3*	1.42	95.3	30129	750	80	14	61	22
"	F3 *C4*	1.42	65.2	44871	750	80	9	28	33
Weinberg (2)	Ex III	26.9	1750.2	482425	100		13	70	19
"	Ex IV	26.9	2451.3	549964.5	100	57	18	86	21
"	Ex V	26.9	954.12	511370.5	100	57	7	36	20
"	Ex VI	1.34	66.08	15000	100	55	10	85	12
"	Ex VIII	1.34	52.82	11850	100	55	8	86	9
Barber	Ex I	0.81	43.98	10800	-[c]	70	11	79	14
"	Ex 9	0.67	48.82	10800	-[c]	70	15	87	17
"	Ex 10	0.54	46.56	10800	-[c]	70	17	83	21
"	Ex 11	0.67	52.06	10800	-[c]	70	16	93	17
Saito	Ex I	2.69	59.93	14400	111	50	5	80	6
"	Ex II	2.69	60.58	14400	111	55	5	81	6
This work	T1 E3	0.2	28.4	5826	350	80	28	94	30
"	T1 E1	0.2	54.78	15422	300	75	55	69	80
"	TS6 E2	0.64	148.4	49464	350	75	46	58	80

a) Weinberg (1): J. Appl. Electrochem. **1991**, *21*, 895–901. Weinberg (2): US 4,478,694. Barber: US 4,517,062. Saito: US 4,270,992. b) T = Table, E = Entry, F = Figure, Ex = Example c) graphite rods were used

6. Inductively-Coupled Mass Spectrometry (ICP-MS) and Inductively-Coupled Optical Emission Spectroscopy (ICP-OES) Measurements

Inductively-coupled mass spectrometry (ICP-MS) and inductively-coupled optical emission spectroscopy (ICP-OES) measurements were performed by following SFS-EN ISO 17294-2:2016 and SFS-EN ISO 11885:2009 standards, respectively. The elemental composition of 0.5 M sulfuric acid solution before and after contact with the graphite electrodes was compared (Table S9) and all values are reported as mg/L. The latter sample was prepared by first crushing a thin sheet of graphite into small pieces. The pieces (2.25 g) were then immersed in 5 mL 0.5 M sulfuric acid for 10 minutes and stirred magnetically with an unused PTFE-coated magnet. Finally, the graphite pieces were separated from the solution by filtration through a glass funnel into glass vials.

Table S9. Elemental composition of 0.5 M sulfuric acid solution before and after contact with the graphite electrodes as determined with ICP-OES and ICP-MS. All values are reported as mg/L.

Entry	Element	Before	After	Difference
1	Al	1.7	1.9	0.2
2	B	12	17	5
3	Ca	0.35	4.6	4.25
4	Fe	0.15	1.1	0.95
5	Cu	0.009	0.029	0.020
6	Mg	0.14	0.22	0.08
7	Na	11	20	9
8	P	0.32	0.30	-0.02
9	Si	7.3	10	2.7
10	Sn	<1	2.1	-
11	Sr	0.0037	.057	0.053
12	Zn	2.6	1.6	-1

7. X-ray Photoelectron Spectroscopy (XPS)

X-ray Photoelectron Spectroscopy (XPS) analysis performed by Thermo Fisher Scientific ESCALAB 250Xi XPS System at the Centre for Material Analysis, University of Oulu (Finland). The monochromatic AlKα radiation (1486.7 eV) operated at 20 mA and 15 kV with X-ray spot size of 900 μm. The high-resolution scan used pass energy of 20 eV while Survey scan used pass energy of 150 eV. The powder samples were put in gold sample holder and O, C, and Au were measured for all samples. The measurement data were analyzed by Avantage V5 program developed by Thermo Fisher Scientific. Charge compensation was carried out by applying the C1s at 284.8 eV as a reference to determine the presented spectra and calibrate the binding energies (BE). The XPS measurements from the pristine (Figures S5–S7) and H_2SO_4-treated graphite (Figures S8–S10) show a 1.8 and 2.0% O-content that is similar to what has been reported for SFG 44 graphite in a separate study.[3] Noteworthy, the O1s spectra are very similar, indicating a similar distribution of surface oxygen groups. Furthermore, in both samples, the C1s spectra displays a dominant peak at 284.8 eV which is tentatively assigned to graphitic carbon.[3] C1s spectra also shows a major peak at 285.2 eV which could originate from asymmetry of the graphitic peak, C-C or C-H bonds, or adventitious hydrocarbons.[3] As for other contributions, 286.8 eV has been assigned both to the alcohol/ether(epoxide) and carbonyl bonds, whereas 288.6 – 289.1 eV (288.7 eV in Table S10) has been assigned to carboxyl and ester groups in the literature.[3] The peak observed at 291.2 eV is probably due to π-π* excitations. In a separate study performed with GO, 284.5 eV was assigned to graphitic carbon, 285.4 eV to sp^3-hybridized carbon, 286.5 eV to hydroxyls and

epoxides, 288.9 eV to carboxyl groups, and 290.4 eV to π-π* excitations.[4] The O1s spectra peaks were assigned tentatively to C=O (531.3 ev), C-O-C (532.7 eV), C-OH (533.8 eV), and COOH (535.3 eV) following literature assignments.[5,6]

Table S10. Overview of XPS-results and tentative assignment of the fitted values.

Entry:	1	2	
Notes:[a]	Non-treated	After H_2SO_4-treatment	Tentative Assignment for C1s[3,4] and O1s[5,6]
C1s / 284.8 eV	0.58	0.57	Graphitic C (C=C)
C1s / 285.2 eV	0.25	0.26	Asymmetry of C=C, C-C, C-H, adventitious hydrocarbons
C1s / 286.6 eV	0.07	0.074	Alcohols, phenols, ethers (C-O)
C1s / 288.7 eV	0.03	0.03	Carboxyl or ester (RO-C=O), (carboxylate)
C1s / 291.2 eV	0.07	0.07	$\pi-\pi$*
O1s / 531.3 eV	0.24	0.25	C=O
O1s / 532.7 eV	0.46	0.45	C-O-C
O1s / 533.8 eV	0.23	0.23	C-OH
O1s / 535.3 eV	0.08	0.07	COOH
O / (C+O)[b]	1.8	2.0	

a) C1s and O1s values are normalized b) O / (C+O) calculated from O1s and C1s atomic-%.

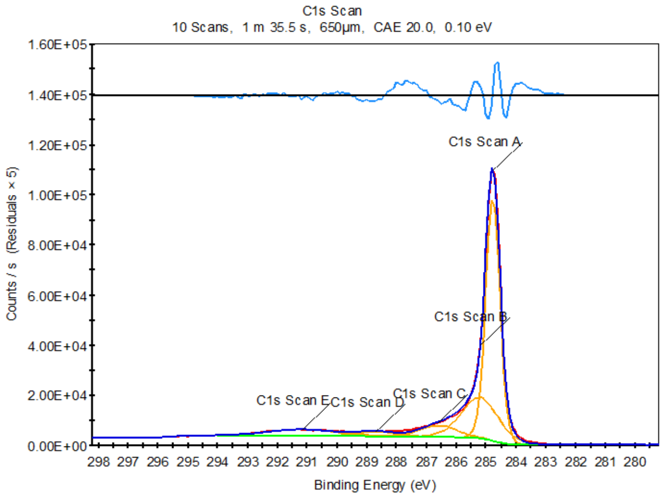

Figure S5. C1s spectra of non-treated graphite.

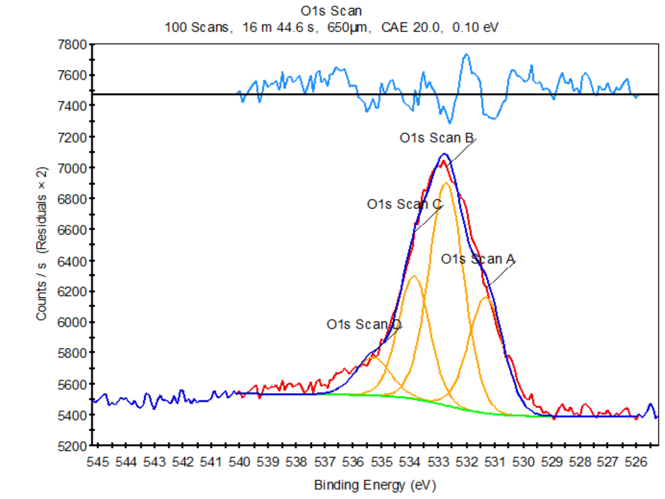

Figure S6. O1s spectra of non-treated graphite.

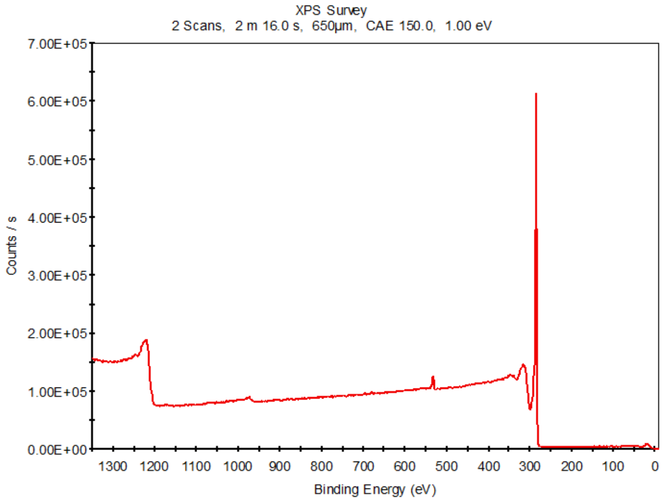

Figure S7. XPS survey spectra of non-treated graphite

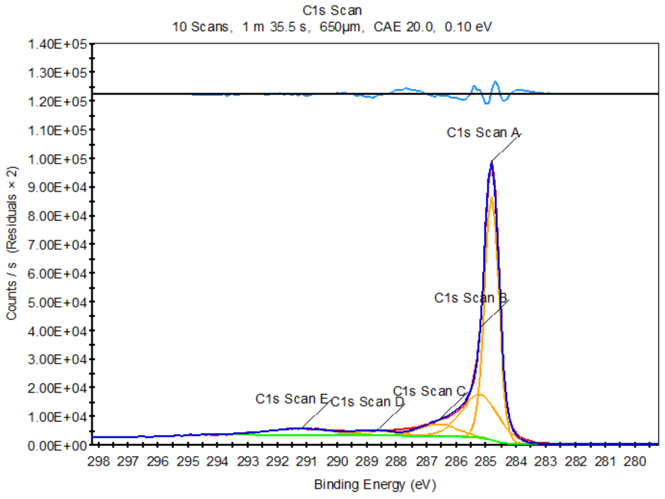

Figure S8. C1s spectra of graphite after treatment with 0.5M H_2SO_4

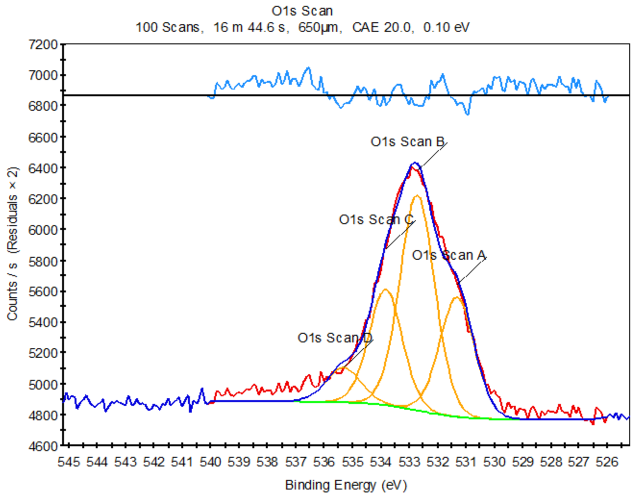

Figure S9. C1s spectra of graphite after treatment with 0.5M H_2SO_4

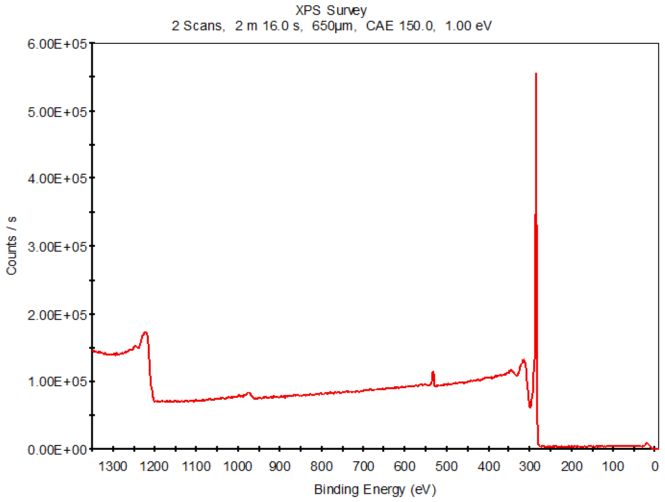

Figure S10. XPS survey spectra of graphite after treatment with 0.5M H_2SO_4

8. Trapping Experiments

Samples and blanks were analyzed by liquid chromatography (Agilent 1260 Infinity High Pressure Liquid Chromatography (HPLC) System (Agilent Technologies, Singapore), coupled to a quadrupole time-of-flight mass spectrometer (6530 Accurate-Mass Q-TOF Agilent Technologies, Santa Clara, USA) operated in positive ionization mode. Analysis was performed by injecting 5 µL sample on a ZORBAX SB-C18 column (Solvent Saver Plus 3.0 × 75 mm, 3.5 Micron, Agilent, USA). A gradient program with water (A) and acetonitrile (B) was run; 0 min; A 80%, B 20%; 5 min, A 80%, B 20%; 20 min, A 50%, B 80%; 30 min, A 50%, B 80%; 45 min A 20%, B 80%; 65 min A 20%, B 80%; 70 min A 5%, B 95%; 80 min, A 5%, B 95%; 85 min A 80%, B 20% ; 100 min A 80%, B 20%, at a flow rate of 0.4 mL min^{-1}. The total run time was 100 min. Two technical replicates were recorded for each sample. The mass accuracy of the instrument using external calibration was specified to be ≤ 3 ppm. Detection was carried out within a mass range of 100-3200 m/z. The analytes were measured in positive ion

mode and the capillary voltage was set to +3500 V. Drying gas flow was set to 12 L min^{-1} with a temperature of 300 °C. Nebulizer, fragmentor, skimmer, and octopole RF set to 25 psi, 150 V, 65 V and 500 V, respectively. Data was collected using Agilent MassHunter Data Acquisition LCMS program. The data treatment was performed using Qualitative Analysis version B.07.00 or B.10.00.

Samples for radical trapping experiments were obtained using standard operation conditions (Section 3), equipped with two sulphuric acid pretreated graphite electrodes. NaCl (0.90 g, 15.4 mmol), TEMPO (0.12 g, 0.8 mmol) and tributylmethylammonium chloride (0.75 g, 3.2 mmol) were dissolved in 15 mL 37 wt-% formaldehyde solution, 7 mL MeOH and 3 mL H_2O. Finally, *N*-cyclohexyl-2-(((2,2,6,6-tetramethylpiperidin-1-yl)oxy)methyl)acrylamide (CHANT) (163 mg, 0.5 mmol) was added to the mixture as a radical scavenger. The mixture was then preheated at 75 °C for 15 minutes and electrolyzed at 350 mA/cm^2 for 41 minutes. The reaction mixture was diluted to 40 mL with water and extracted twice with 20 mL DCM. The results were compared to a blank sample which was obtained by heating a mixture of CHANT (20 mg), MeOH (1 mL) and formalin (1 mL) for 1 h at 75 °C. The resulting solution was extracted to DCM (2 mL) after having cooled down to room temperature. HPLC / ESI-HR-MS traces of the reaction mixture are shown in Section 12.

9. Mechanistic Experiments with Methanol-d_4 and Formaldehyde-d_2

We performed additional mechanistic experiments with CD_3OD and CD_2O. Earlier control experiments with non-labelled starting materials indicated that, in the presence of TEMPO, formaldehyde concentration is higher than in its absence (Table S11, Entry 1 vs. 2, Entry 3 vs. 4, Entry 5 vs. 6) after the reaction. *If* the increase in formaldehyde concentration would be solely from TEMPO-mediated oxidation of methanol to formaldehyde, current efficiencies of 43 and 19% would be obtained in high current efficiency (Table 1, Conditions B) and high yield (Table 1, Conditions A) reaction conditions, respectively. Noteworthy, this consideration does not take into account unmediated methanol oxidation, that can take place as well (Table S11, Entry 6), and decomposition reactions or evaporation (Table 1, Entry 5). In addition, electrolytic formaldehyde reduction to methanol further complicates the interpretation of the obtained data. Furthermore, TEMPO has been successfully used as a mediator for oxidation of alcohols in the literature.[7–10] Based on these considerations and existing literature, we hypothesised that TEMPO could act as a mediator for anodic oxidation of methanol into formaldehyde.

Table S11. Control experiments performed with non-deuterated starting materials.

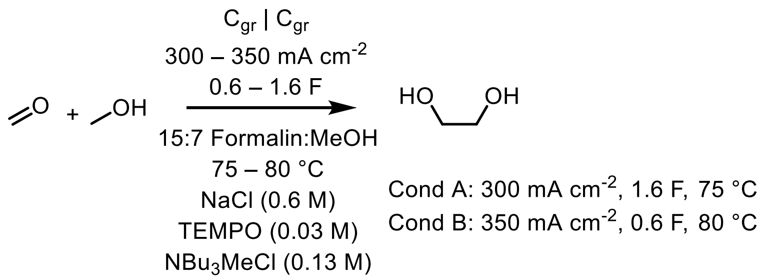

Entry:	Notes:	FA before electrolysis (n / mmol)	FA after electrolysis (n / mmol)	ΔFA (n / mmol)	Hypothetical TEMPO c.e. (%)
1	Table 1, Entry 1<br>Conditions A	200	23.2	14.9	19
2	Table 1, Entry 2<br>Conditions A, No TEMPO	200	8.3	-	-
3	Table 1, Entry 3<br>Conditions B	200	113	13.0	43
4	Table 1, Entry 4<br>Conditions B, No TEMPO	200	100	-	-
5	Table 1, Entry 6<br>Conditions B, No FA	0	8.4	4.9	16
6	Table 1, Entry 6,<br>Conditions B, No TEMPO, No FA	0	3.5	-	-

For scrutinizing this hypothesis further we first performed experiments without formaldehyde in CH_3OH, CD_3OD, and CH_3OH:CD_3OD (Table S12). In these trials, the reactions were performed in PTFE screening cells,[11] which slightly decreased the current efficiencies in comparison to a larger glass cell. The reactions were performed both with 0.6 F and 1.6 F at 75 °C and with 300 mA cm^{-2} current density. These experiments show that undeuterated formaldehyde is obtained in 1.5 – 2.0 higher yield than the d_2-formaldehyde (Table S12). Furthermore, small amounts of EG were also detected in these experiments.

We also performed similar studies with CH_2O, CD_2O, and CH_2O:CD_2O in the presence of various amounts CH_3OH and CD_3OD (Table S13). Noteworthy, CH_3OH:CD_3OD ratio in these cases is more difficult to control as the CH_2O contains methanol as a stabilizer (commercial formalin solution) and because CD_2O was prepared with an anodic oxidation reaction from CD_3OD. The latter is also the reason why we performed these

reactions at a lower concentration of 0.7 M in a PTFE screening cell, as we were not able to obtain 8 M solution with this synthetic strategy. Interestingly, when the reaction is performed with deuterated formaldehyde (Table S13, Entry 2) in the presence of CH_3OH, EG is obtained in 12:88 H:D ratio with concomitant formation of formaldehyde at 25% current efficiency. Furthermore, when the reaction is performed with commercial formalin solution at 8 M concentration and 25 mL scale with CD_3OD as an additive (Table S13, Entry 4), EG is obtained in 50% yield in a 97:3 H:D ratio together with concomitant formation of CD_2O. By using the ratio of 1.5 CH_2O:CD_2O that was obtained in the methanol oxidation studies (Table S12), we estimate that 1.6 mmol and 6.6 mmol of total non-labelled and deuterated formaldehyde could be formed in Entry 2 and Entry 4, respectively. The former value would correspond to 41% current efficiency, whereas the latter would constitute of 8% (i.e., 42%) out of the 19% of the hypothetical current efficiency increase with TEMPO (Table S11, Entry 1). We want to stress that these considerations do not take into account formaldehyde evaporation/decomposition, possible formaldehyde reduction to methanol in high yield conditions and CH_3OH:CD_3OD ratio effects in the anodic oxidation. However, at the same time, we clearly see that i) in the presence of TEMPO, the concentration of formaldehyde after the reaction is higher (Table S11), and ii) methanol from the reaction mixture is converted into both formaldehyde and ethylene glycol (Tables S12 – S13). We propose that TEMPO has a positive effect on the anodic oxidation of methanol, but at the current state, we cannot rule out any other mechanistic TEMPO pathways that could contribute towards more successful EG synthesis.

Table S12. Mechanistic studies on anode reaction with methanol and methanol-d4 with TEMPO, at 75 °C

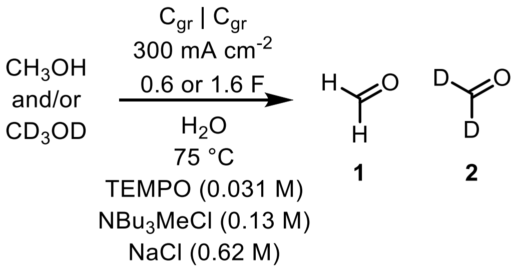

Entry	SM[a]	Charge (F)	1 c.e (%)[b]	2 c.e. (%)[c]
1	MeOH	0.6	21	-
2	MeOD-d4	0.6	-	14
3	1:1 MeOH:MeOD-d4	0.6	13	8
4	MeOH	1.6	16	-
5	MeOD-d4	1.6	-	8
6	1:1 MeOH:MeOD-d4	1.6	6	4

[a] Volume of starting material was 2.5 mL, mixed with 2.5 mL water. [b] Yields determined by ^1H NMR using dimethyl sulfone ($DMSO_2$) as an internal standard. [c] Yields determined by ^2H NMR using acetonitrile-d_3 as an internal standard.

Table S13. Mechanistic studies on with formaldehyde and formaldehyde-d_2 with TEMPO at 75 °C

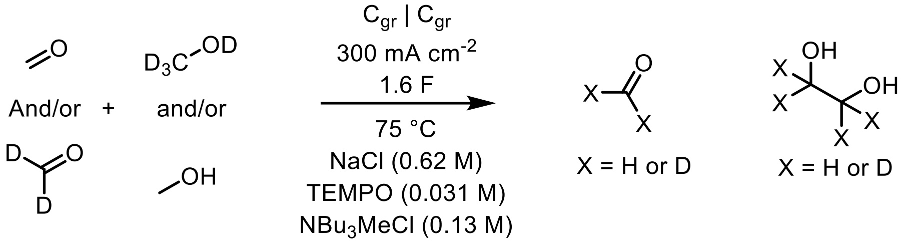

Entry	FA (n / mmol)	FA-d_2 (n / mmol)	MeOH (mmol)	CD_3OD (mmol)	EG yield % (H:D)[a][b]	FA mmol (H:D)[a][b]
1	4.85	-	1	49	3.4 (100:0)	2 (100:0)
2	-	4.85	49	39	7 (12:88)	4.62 (21:79)
3	2.425	2.425	25	25	13.2 (44:56)	3.17 (39:61)
4	200	-	47	170	49.7 (97:3)	24.3 (89:11)

[a] Yields determined by ^1H NMR using dimethyl sulfone ($DMSO_2$) as an internal standard. [b] Yields determined by ^2H NMR using acetonitrile-d_3 as an internal standard.

9.1 Electrosynthesis of Formaldehyde-d_2.

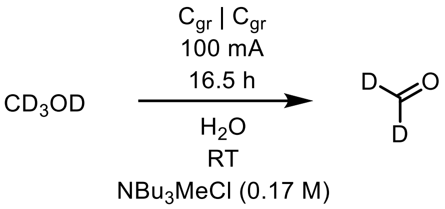

Scheme S2. Electrosynthesis of formaldehyde-d_2 from methanol-d_4

Formaldehyde-d_2 was synthesized by electro-oxidation of methanol-d_4 in a PTFE screening cell. First, 200 mg NBu_3MeCl was dissolved in 2.5 mL methanol-d_4 and 2.5 mL water. Then, two sulfuric acid treated graphite electrodes were attached to the cell, and the solution was electrolyzed for 16.5 h with 100 mA current (59 mA cm^{-2}). After the electrolysis, the solution was diluted to 5.0 mL with water, and the amount of formaldehyde-d_2 was calculated to be 4.85 mmol based on ^2H NMR spectra.

9.2 Characterization of ^{2}H-Labelled Compounds

All the products of the labeling experiment were analyzed with NMR. First, non-deuterated products were analyzed according to the standard ethylene glycol and formaldehyde quantification method. Second, deuterated products were assigned and analysed using ^{1}H and ^{2}H NMR. Deuterated ethylene glycol was analyzed directly from 600 µL of the solution after electrolysis or from the same sample as formaldehyde-d_2, which was analyzed from a solution consisting of 300 µL reaction mixture and 300 µL of sodium bisulphite (38-40 % in H_2O). Acetonitrile-d_3 (5.0 µL) was added to both samples as an internal standard for ^{2}H NMR quantification. ^{2}H NMR spectra were acquired with a zg2h pulse sequence using d1 relaxation delay of 25 s, while methanol-d_4 was used for locking and shimming of the sample. Both ethylene glycol-d_2 and ethylene glycol-d_4 were observed, however, the two compounds could not be differentiated due to overlapping peaks. The spectra and the assignments of the peaks are presented in Section 11.

10. Synthesis of CHANT

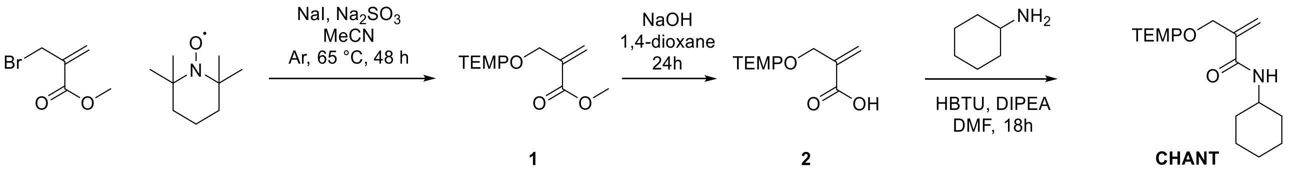

Scheme S3. Synthesis of *N*-cyclohexyl-2-(((2,2,6,6-tetramethylpiperidin-1-yl)oxy)methyl)acrylamide.

CHANT was prepared according to a literature procedure.[12] (2,2,6,6-Tetramethylpiperidin-1- yl)oxyl (1.88 g, 12.0 mmol, 1.2 equiv.), NaI (3.00 g, 20.0 mmol, 2.0 equiv.) and Na_2SO_3 (3.78 g, 30.0 mmol, 3.0 equiv.) were weighted into a dried 200 mL flask. The solids were dissolved in MeCN (100 mL) under Ar, followed by addition of methyl 2-(bromomethyl)acrylate (1.79 g, 10.0 mmol, 1.0 equiv.). The solution was then mixed at 65 °C under Ar for 48 h. MeCN was removed in a vacuum. The resulting oil was dispersed in water (100 mL) and extracted by EtOAc (3x 100 mL). The organic phase was dried with Na_2SO_4 and filtered. The solution was evaporated to dryness in a vacuum affording red crude oil, which was purified using column chromatography (silica gel, 5/1 Cyclohexane/EtOAc), affording methyl 2-(((2,2,6,6-tetramethylpiperidin-1-yl)oxy)methyl) acrylate (Scheme S3, **1**) as a clear oil (1.46 g, 48%). Then, 1,4-Dioxane (40 mL) and aqueous NaOH (40 mL, 1 M) were then added to the **1** (1.46 g, 5.7 mmol), and the resulting solution was stirred for 24 h. The solution was then acidified with aqueous HCl (40 mL, 2 M) and extracted with EtOAc (first 3x 50 mL, then 2x 100 mL). The combined organic phases were dried with Na_2SO_4 and evaporated to dryness, giving 2-(((2,2,6,6-tetramethylpiperidin-1-yl)oxy)methyl)acrylic acid **2** (Scheme S3, **2**) as a white solid (0.81 g, 59%), which was used without further purification.

In the final step, cyclohexylamine (0.23 mL, 2 mmol), **2** (0.48 g, 2 mmol), HBTU (0.83 g, 2.2 mmol) and DIPEA (0.70 mL, 4 mmol) were dissolved in DMF (10 mL) and stirred for 18 h at room temperature. Volatiles were then removed in a vacuum, and saturated $NaHCO_3$ (20 mL) was added to the residue. The resulting mixture was stirred for 5 minutes, after which it was extracted with EtOAc (3x 20 mL). The combined organic phases were washed with saturated brine (3x 20 mL) and dried with Na_2SO_4. The solvent was removed in a vacuum, leaving the crude product as a brown oil. The crude was purified using column chromatography (silica gel, 3/1 Cyclohexane/EtOAc) affording *N*-cyclohexyl-2-(((2,2,6,6-tetramethylpiperidin-1-yl)oxy)methyl)acrylamide (Scheme S3, **CHANT**) as a white solid (0.41 g, 64%).

^{1}H-NMR ($CDCl_3$, 500 MHz) δ: 6.61 (d, J = 7.6 Hz, 1H), 6.08 (d, J = 1.8 Hz, 1H), 5.48 (m, 1H), 4.48 (d, J = 1.1 Hz, 2H), 3.84 (m, 1H), 2.03 – 1.93 (m, 2H), 1.74 (dt, J = 14.4, 3.8 Hz, 2H), 1.67 – 1.52 (m, 2H), 1.52 – 1.45 (m, 4H), 1.44 – 1.31 (m, 3H), 1.23 – 1.04 (m, 15H).

^{13}C-NMR ($CDCl_3$, 125 MHz) δ: 165.9, 139.9, 123.9, 77.7, 60.0, 48.2, 39.7, 33.4, 33.1, 25.6, 25.0, 20.4, 17.0.

11. NMR-traces

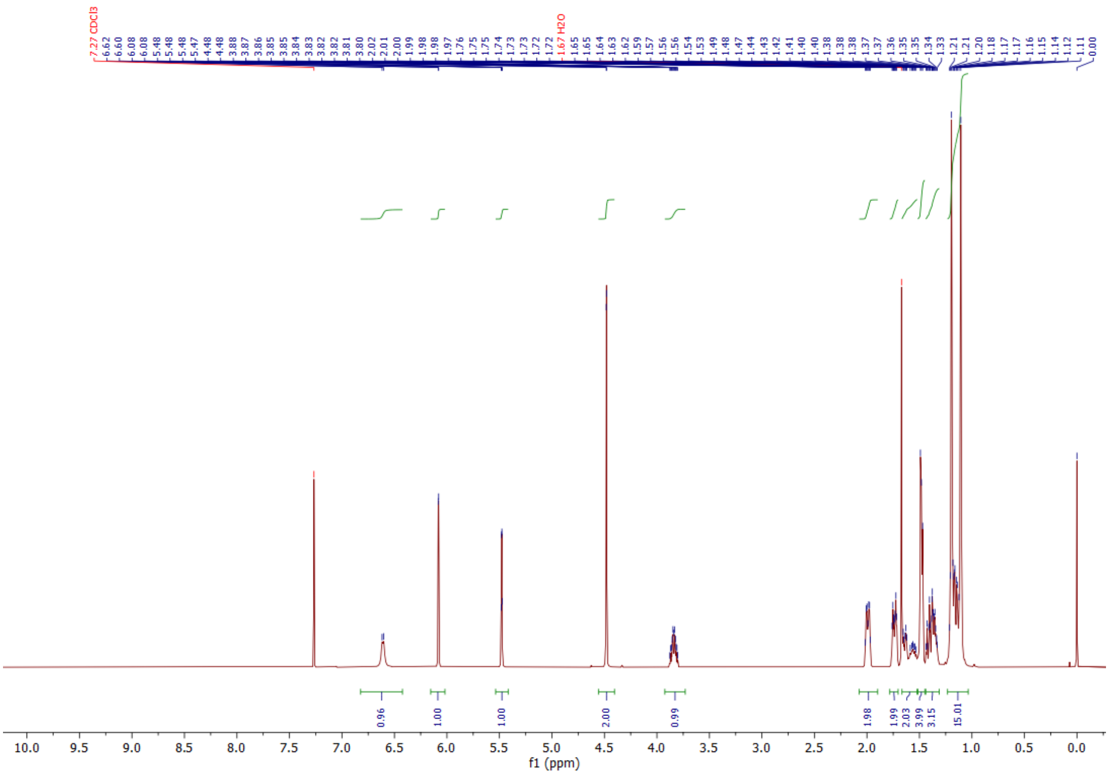

Figure S11. ^{1}H-NMR spectrum of CHANT in $CDCl_3$

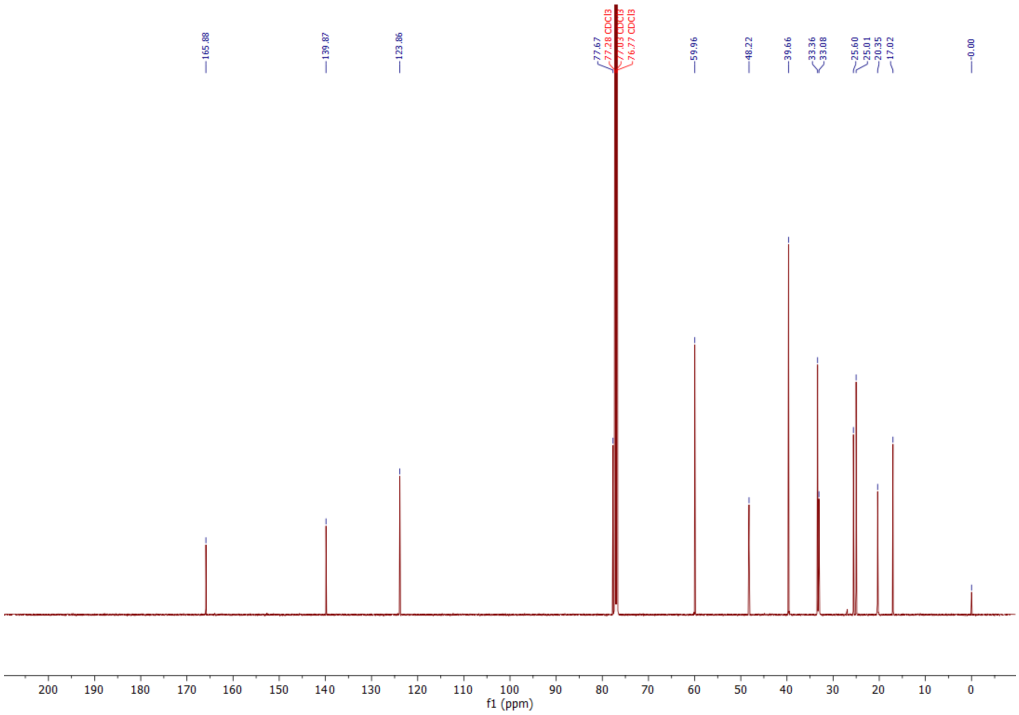

Figure S12. ^{13}C-NMR spectrum of CHANT in $CDCl_3$

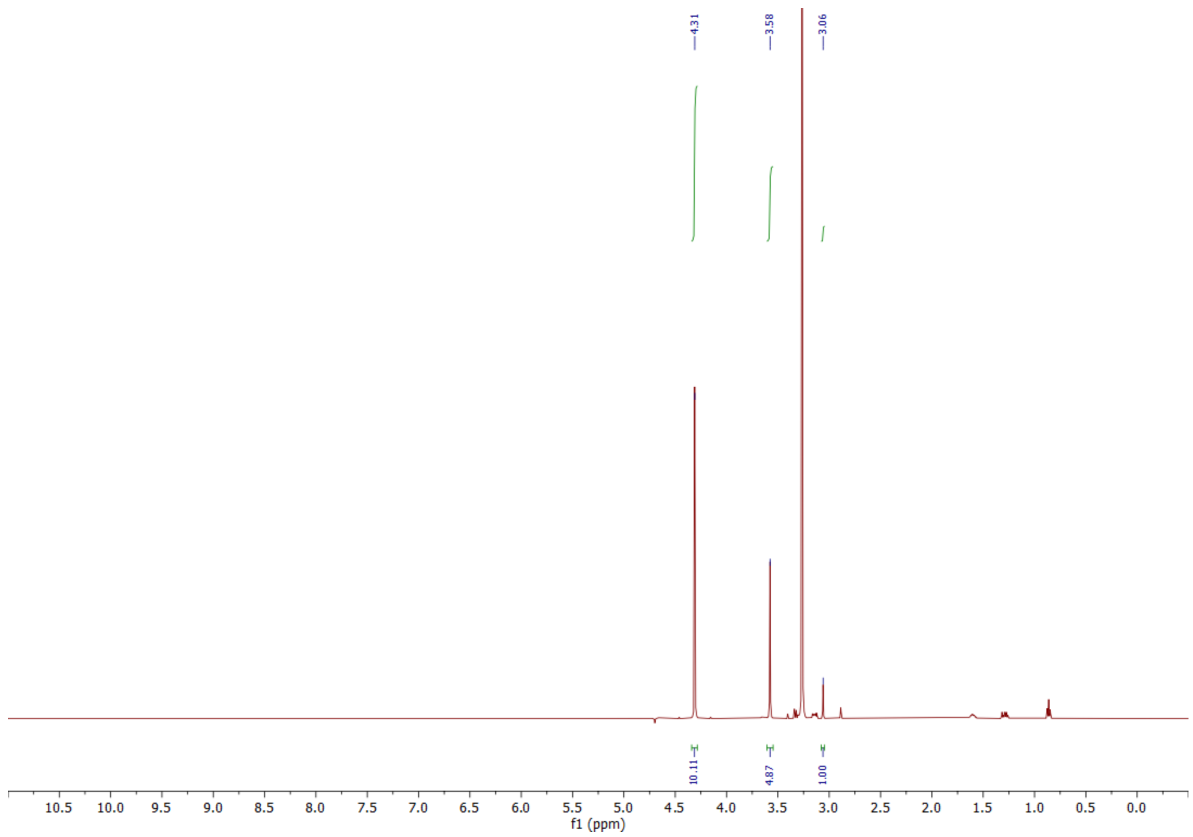

Figure S13. ^{1}H-NMR spectrum of the quantification sample from the reaction mixture with high current efficiency EG synthesis (Table S3, Entry 4).

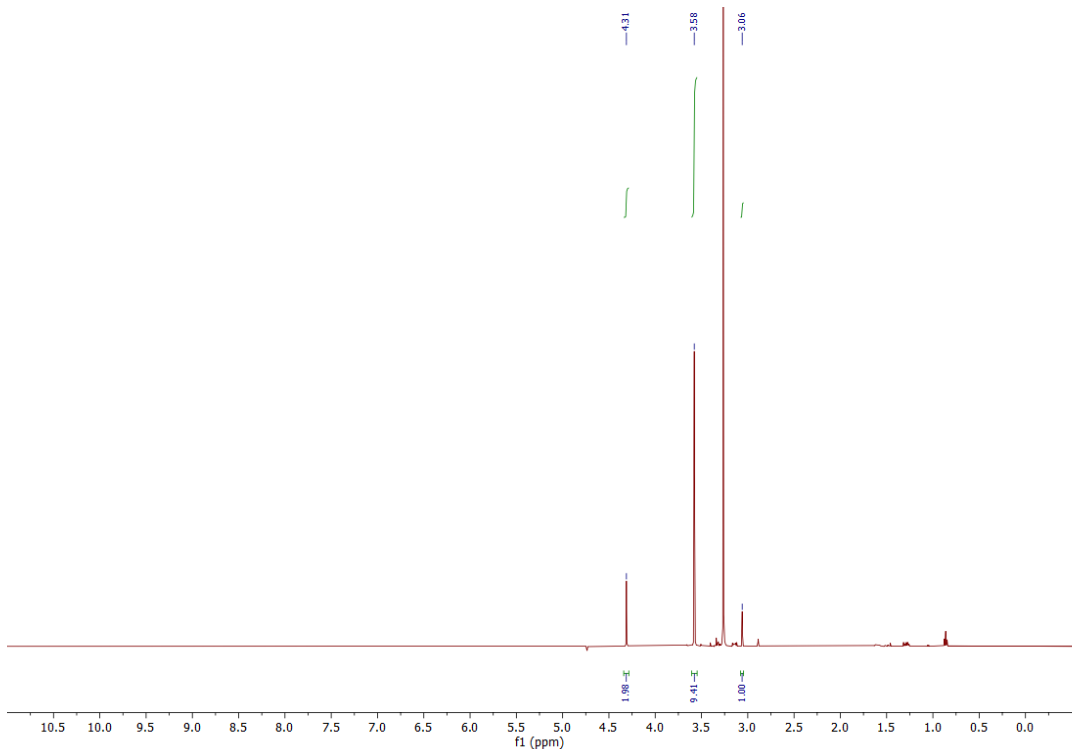

Figure S14. ^{1}H-NMR spectrum of the quantification sample from the reaction mixture with high yield EG synthesis (Table S6, Entry 11).

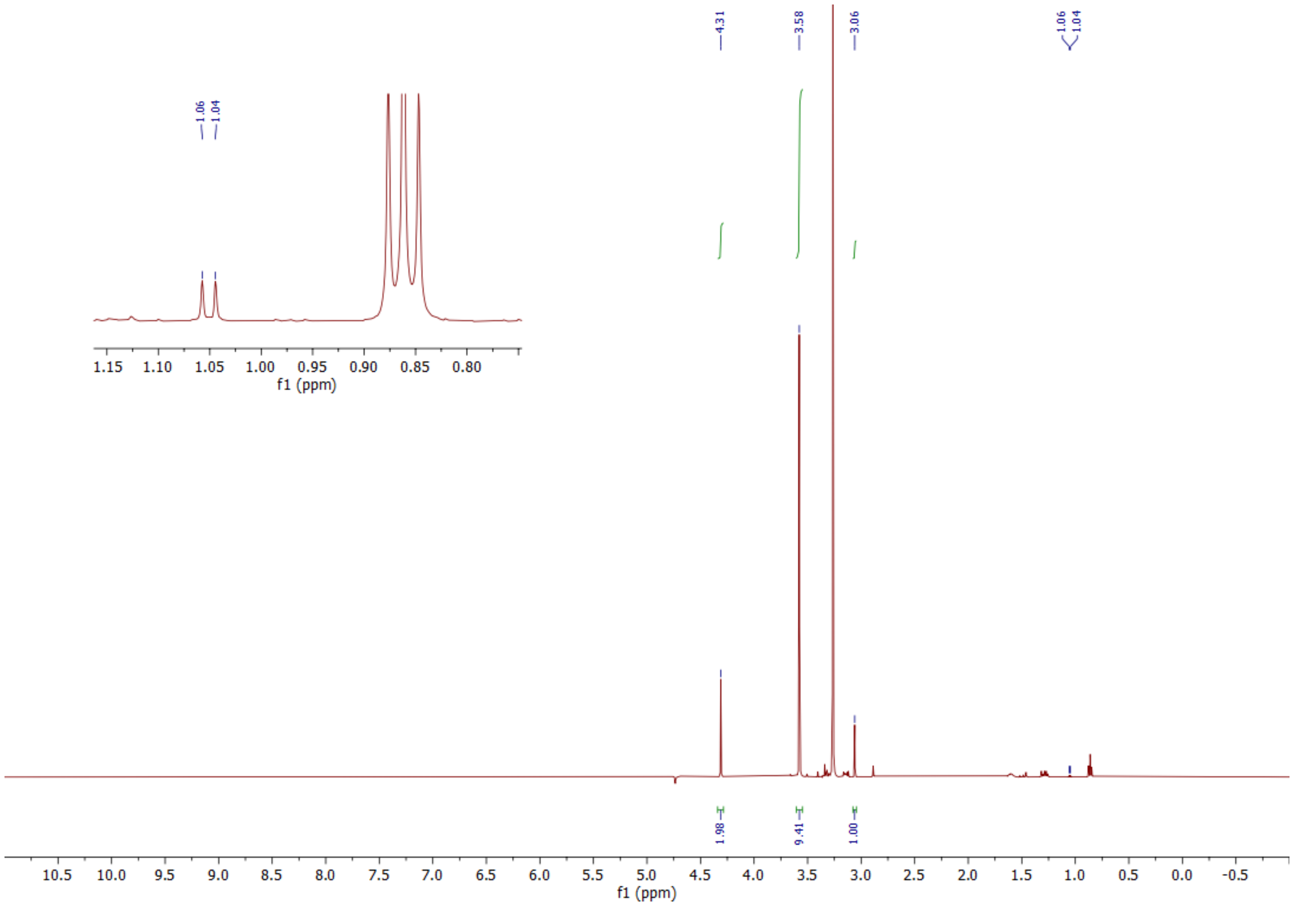

Figure S15. ^{1}H-NMR spectrum of the reaction mixture with high yield EG synthesis (Table S6, Entry 11), showing possible propylene glycol peak at 1.05 ppm.

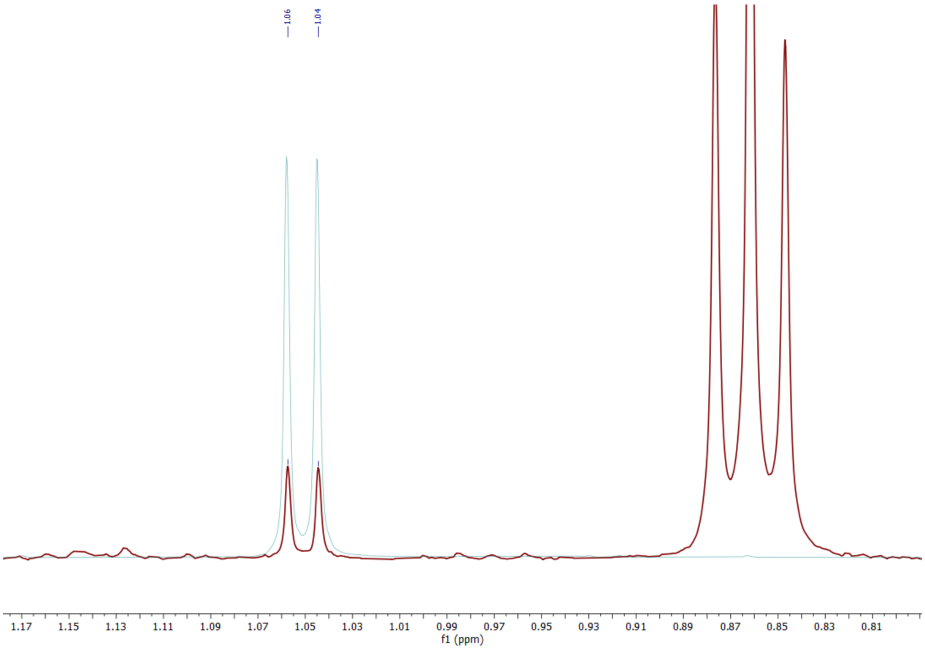

Figure S16. Overlay of ^{1}H-NMR spectra of the reaction mixture (red) and the same sample spiked with 10 µL 1,2-propylene glycol (blue).

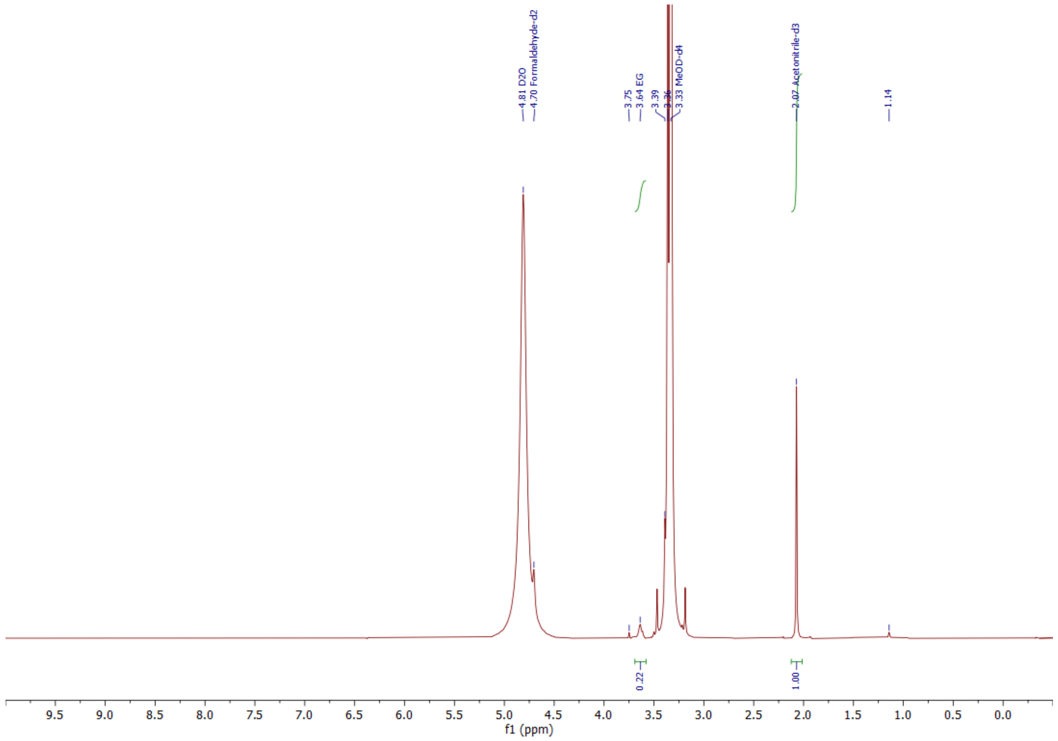

Figure S17. Representative example of ^{2}H-NMR spectrum of the reaction mixture (neat) from the mechanistic experiment showing deuterated formaldehyde (4.70 ppm) and ethylene glycol (3.64 ppm) peaks.

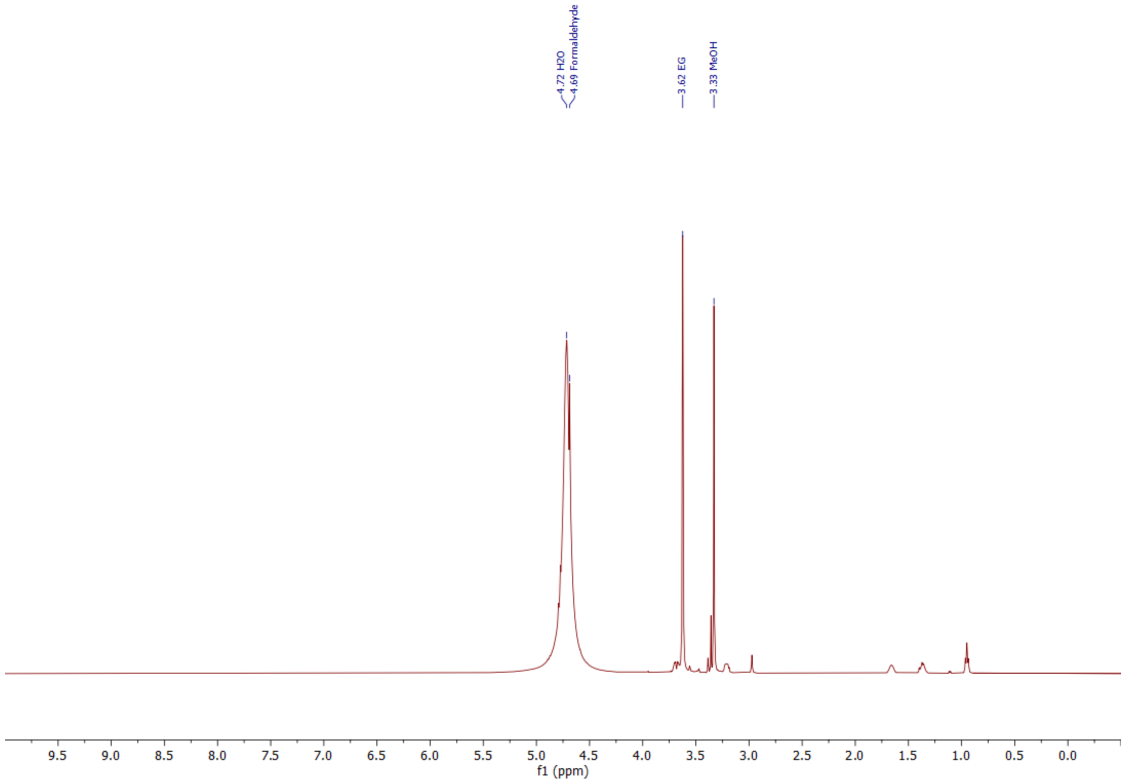

Figure S18. ^{1}H-NMR spectrum of the reaction mixture (neat) from the mechanistic experiment showing formaldehyde (4.69 ppm) and ethylene glycol (3.62 ppm) peaks.

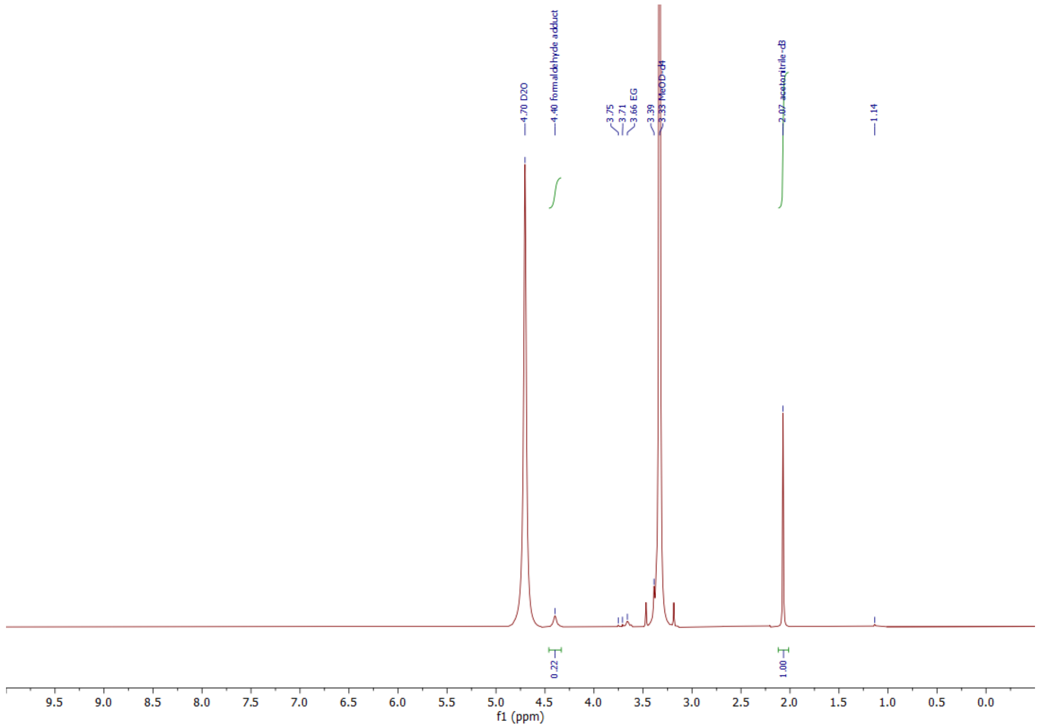

Figure S19. Representative example of ^{2}H-NMR spectrum of a 1:1 solution of reaction mixture and sodium bisulphite (38-40 % in H_2O) from the mechanistic experiment showing deuterated formaldehyde-bisulfite adduct (4.40 ppm) and ethylene glycol (3.66 ppm).

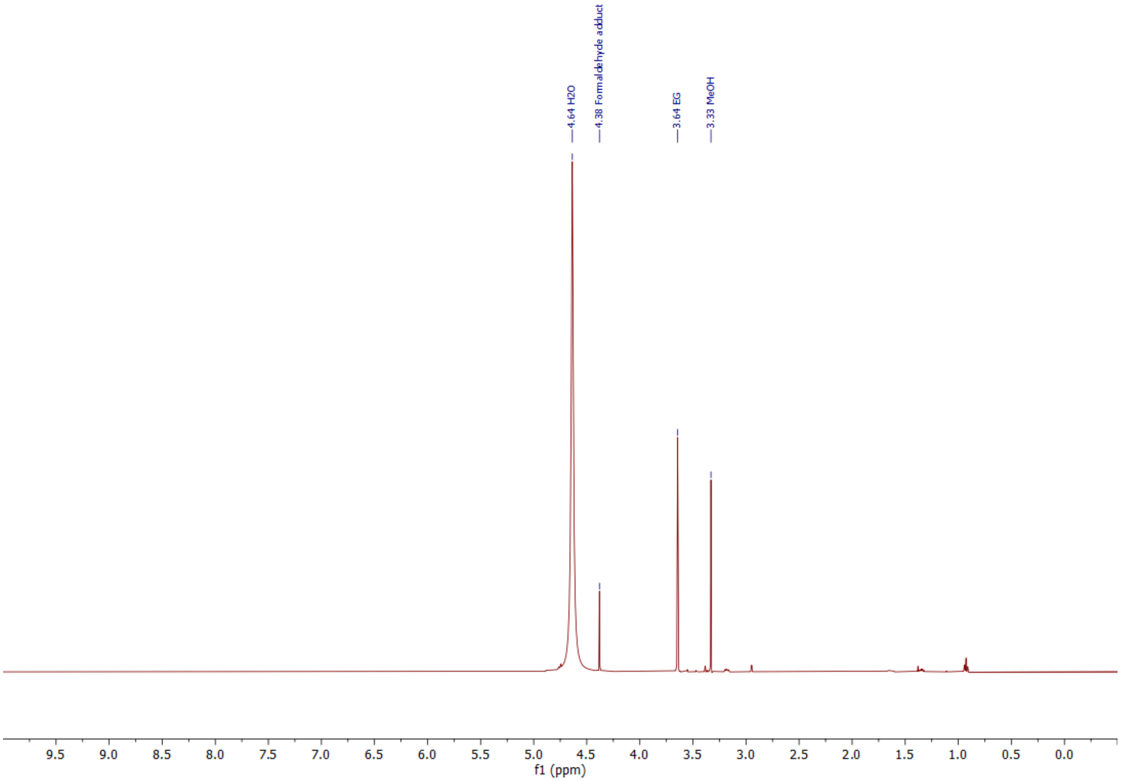

Figure S20. ^{1}H-NMR spectrum of a 1:1 solution of reaction mixture and sodium bisulphite (38-40 % in H_2O) from the mechanistic experiment showing formaldehyde-bisulfite adduct (4.38 ppm).

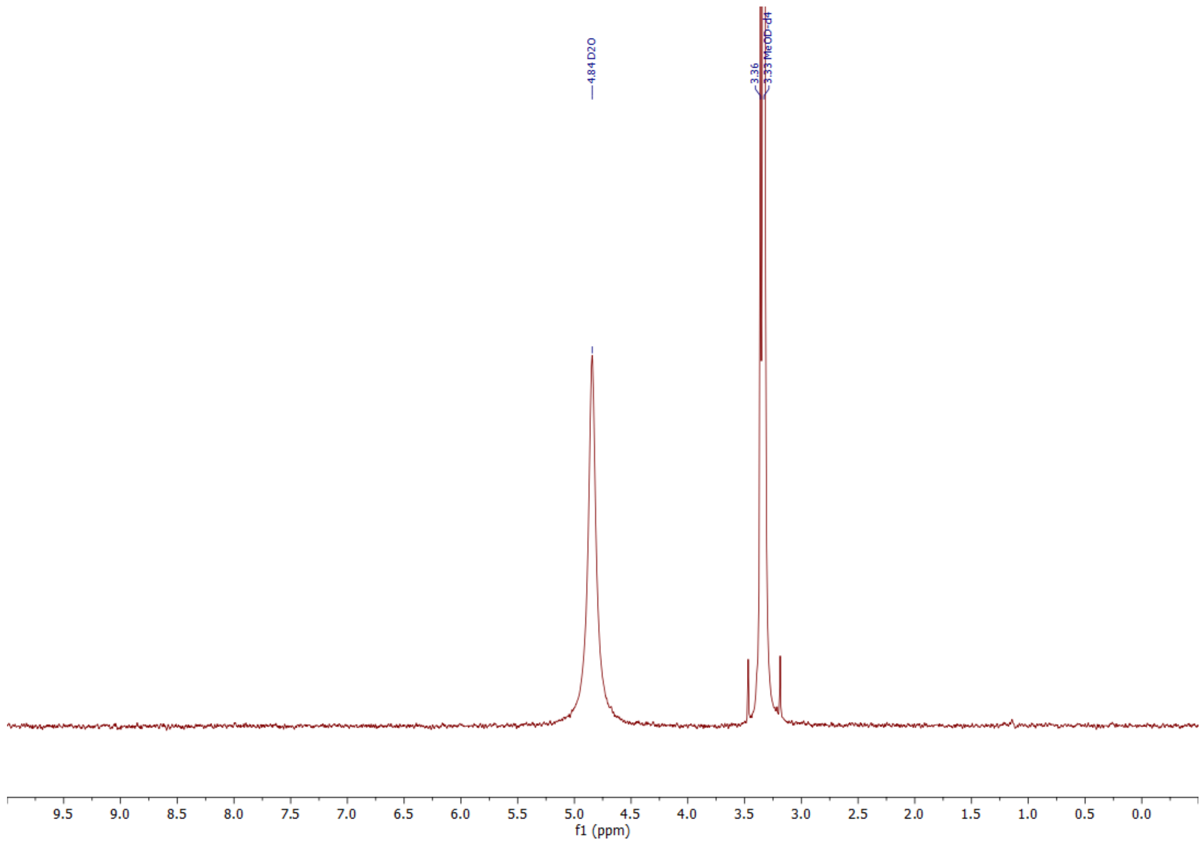

Figure S21. Blank ^2H-NMR spectrum from the reaction mixture (neat) of Table S6, Entry 11, diluted with methanol-d_4.

12. HPLC / ESI-HR-MS traces

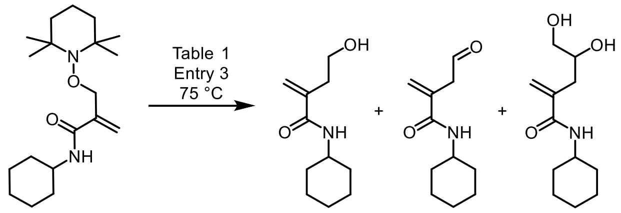

Scheme S4. Experiments in the presence of a CHANT-type radical trap.

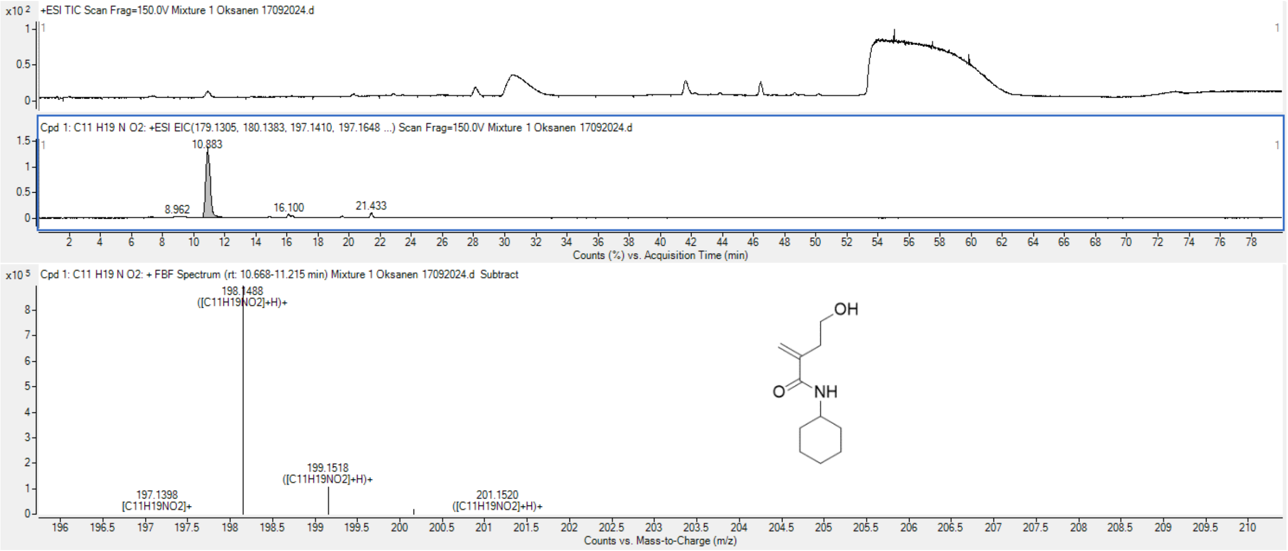

Figure S22. HPLC/HR-ESI-MS trace of *N*-cyclohexyl-4-hydroxy-2-methylenebutanamide from the reaction according to Scheme S4. Mass calculated for $[C_{11}H_{19}NO_2 + H]^+$ 198.1489 found: 198.1488 (Δ = 0.01 ppm).

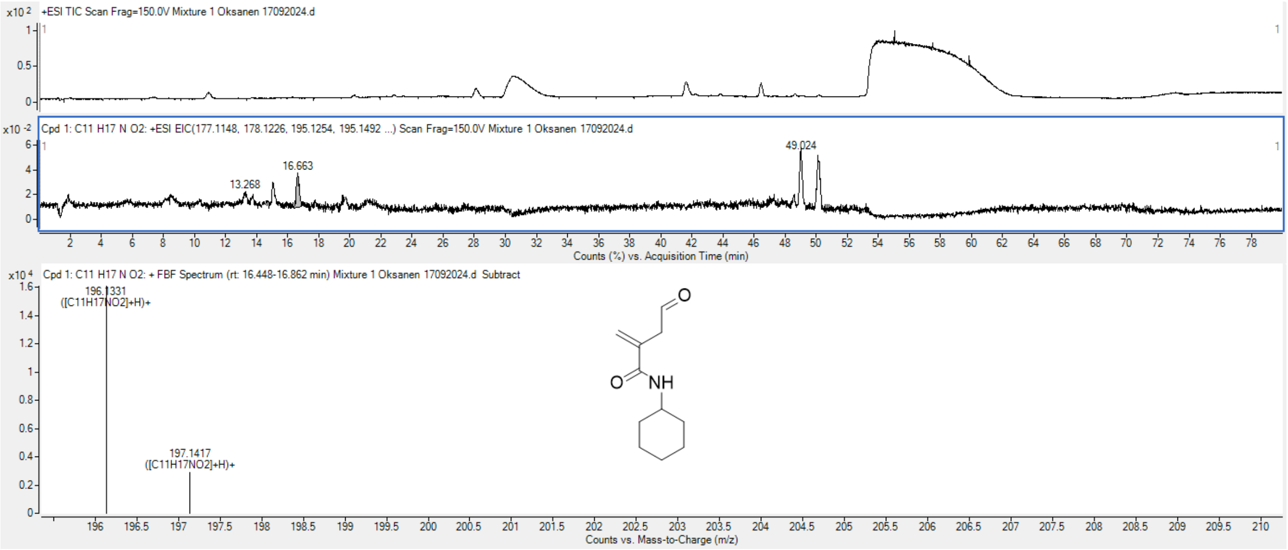

Figure S23. HPLC/HR-ESI-MS trace of *N*-cyclohexyl-2-methylene-4-oxobutanamide from the reaction according to Scheme S4. Mass calculated for $[C_{11}H_{17}NO_2 + H]^+$ 196.1332 found: 196.1331 (Δ = 3.11 ppm).

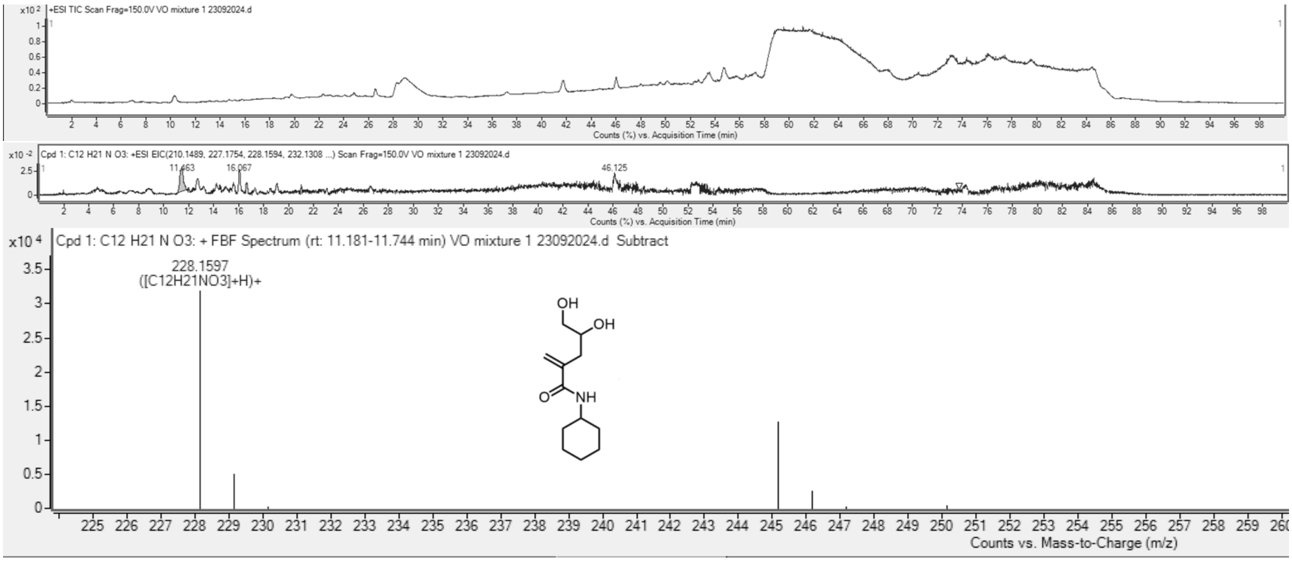

Figure S24. HPLC/HR-ESI-MS trace of *N*-cyclohexyl-4,5-dihydroxy-2-methylenepentanamide from the reaction according to Scheme S4. Mass calculated for $[C_{12}H_{21}NO_3 + H]^+$ 228.1594 found: 228.1597 (Δ = -0.3 ppm)

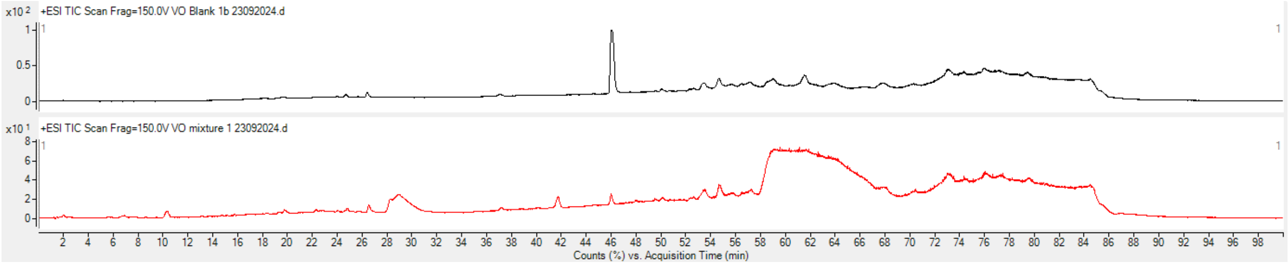

Figure S25. HPLC/HR-ESI-MS from the reaction according to Scheme S4 in the absence of electricity (up) compared to the reaction with constant current (bottom).

13. References

[1] M. Linden, S. Hofmann, A. Herman, N. Ehler, R. M. Bär, S. R. Waldvogel, *Angew. Chem. Int. Ed.* **2023**, *62*, e202214820.

[2] T. Chatterjee, E. Boutin, M. Robert, *Dalton Trans.* **2020**, *49*, 4257–4265.

[3] R. I. R. Blyth, H. Buqa, F. P. Netzer, M. G. Ramsey, J. O. Besenhard, P. Golob, M. Winter, *Appl. Surf. Sci*. **2000**, *167*, 99–106

[4] R. Muzyka, M. Kwoka, Ł. Smędowski, N. Díez, G. Gryglewicz, *New Carbon Mater*. **2017**, *32*, 15–20.

[5] P. W. Albers, V. Leich, A. J. Ramirez-Cuesta, Y. Cheng, J. Hö, S. F. Parker, *Mater. Adv.* **2022**, *3*, 2810.

[6] A. Ashraf, S. A. Dastgheib, G. Mensing, M. A. Shannon, *J. Supercrit. Fluids*. **2013**, *76*, 32–40.

[7] M. Rafiee, B. Karimi, S. Alizadeh, *ChemElectroChem* **2014**, *1*, 455–462.

[8] W. Zhang, P. Chen, S. Chen, Y. Wei, *J. Electroanal. Chem*. **2018**, 815, 130–133.

[9] J. T. Hill-Cousins, J. Kuleshova, R. A. Green, P. R. Birkin, D. Pletcher, T. J. Underwood, S. G. Leach, R. C. D. Brown, *ChemSusChem* **2012**, *5*, 326–331.

[10] P. J. L. Broersen, J. J. N. Koning, G. Rothenberg, A. C. Garcia, *ChemSusChem* **2024**, *17*, e202400582

[11] C. Gütz, B. Klöckner, S. R. Waldvogel Org. Process Res. Dev. **2016** *20 (1)*, 26-32

[12] P. J. H. Williams, G. A. Boustead, D. E. Heard, P. W. Seakins, A. R. Rickard, V. Chechik, *J. Am. Chem. Soc.* **2022**, *144*, 15969–15976.
